# The *rnc* Gene Promotes Exopolysaccharide Synthesis and Represses the *vicRKX* Gene Expressions via MicroRNA-Size Small RNAs in *Streptococcus mutans*

**DOI:** 10.3389/fmicb.2016.00687

**Published:** 2016-05-10

**Authors:** Meng-Ying Mao, Ying-Ming Yang, Ke-Zeng Li, Lei Lei, Meng Li, Yan Yang, Xiang Tao, Jia-Xin Yin, Ru Zhang, Xin-Rong Ma, Tao Hu

**Affiliations:** ^1^State Key Laboratory of Oral Diseases, West China Hospital of Stomatology, Sichuan UniversityChengdu, China; ^2^Department of Dentistry, Yan'an Hospital Affiliated to Kunming Medical UniversityKunming, China; ^3^Chengdu Institute of Biology, Chinese Academy of SciencesChengdu, China; ^4^Department of Endodontics and Operative Dentistry School of Stomatology, Capital Medical UniversityBeijing, China

**Keywords:** *rnc* gene, *vicRKX*, post-transcriptional regulation, microRNA-size small RNAs, exopolysacchrides, *Streptococcus mutans*, dental caries

## Abstract

Dental caries is a biofilm-dependent disease that largely relies on the ability of *Streptococcus mutans* to synthesize exopolysaccharides. Although the *rnc* gene is suggested to be involved in virulence mechanisms in many other bacteria, the information regarding it in *S. mutans* is very limited. Here, using deletion or overexpression mutant assay, we demonstrated that *rnc* in *S. mutans* significantly positively regulated exopolysaccharide synthesis and further altered biofilm formation. Meanwhile, the cariogenecity of *S. mutans* was decreased by deletion of *rnc* in a specific pathogen-free (SPF) rat model. Interestingly, analyzing the expression at mRNA level, we found the downstream *vic* locus was repressed by *rnc* in *S. mutans*. Using deep sequencing and bioinformatics analysis, for the first time, three putative microRNA-size small RNAs (msRNAs) targeting *vicRKX* were predicted in *S. mutans*. The expression levels of these msRNAs were negatively correlated with *vicRKX* but positively correlated with *rnc*, indicating *rnc* probably repressed *vicRKX* expression through msRNAs at the post-transcriptional level. In all, the results present that *rnc* has a potential role in the regulation of exopolysaccharide synthesis and can affect *vicRKX* expressions via post-transcriptional repression in *S. mutans*. This study provides an alternative avenue for further research aimed at preventing caries.

## Introduction

Dental caries is one of the most prevalent and costly biofilm-dependent oral infectious diseases, affecting the majority of the world's population (Selwitz et al., 2007). *Streptococcus mutans* is considered to be the principal pathogen responsible for dental caries (Takahashi and Nyvad, 2008). The ability to synthesize exopolysaccharides, which contribute to the adhesion and formation of tenacious biofilms on tooth surfaces, is recognized as one of the most important virulence factors related to dental caries formation (Nobbs et al., 2009; Bowen and Koo, 2011). As exopolysaccharide is an important virulence factor, exopolysaccharide synthesis-related genes are optimal targets for the development of anti-caries compounds (Chau et al., 2015; Zhang et al., 2015). Among these genes, *vicRKX*, which are known to encode the VicRK signal transduction system (TCS), are crucial to the pathogenicity of *S. mutans*, especially for exopolysaccharide formation (Senadheera et al., 2005, 2007). Basically, the *vicRKX* positively regulate exopolysaccharide production by VicR specifically binding to the promoter regions of genes responsible for the synthesis of glucans and fructans (Senadheera et al., 2005).

The *rnc* gene is recognized as the encoding gene of ribonuclease III (RNase III) (March et al., 1985; Watson and Apirion, 1985). The double-strand-specific endonuclease RNase III is highly conserved in bacteria and eukaryotes and has a critical role in physiology regulation, where it can promote mRNA maturation (Viegas et al., 2011). Much more studies have showed that *rnc* can be instrumental in virulence mechanisms in bacteria (Haddad et al., 2013; Hotto et al., 2015). However, the information regarding *rnc* in *S. mutans* is very limited. Previously, we investigated the genome of *S. mutans* (accession no. AE014133) (Ajdic et al., 2002) and identified an interesting phenomenon: the *rnc* gene is located on upstream of *vicRKX* tricistronic operon. It is suggested that genes located nearby are functionally related and make up a part of a metabolic pathway (Okuda et al., 2007). Thus, we hypothesize that *rnc* may be involved in the regulation of exopolysaccharide synthesis.

Over the last decade, studies of RNA-mediated regulation by small noncoding RNA molecules in eukaryotes and prokaryotes have demonstrated the major roles these molecules play in post-transcriptional regulation in various biological processes (Bartel, 2004; Archambaud et al., 2013). MicroRNAs (miRNAs), known as a special kind of small noncoding RNAs, are endogenous ~23-nt RNAs that can play important gene-regulatory roles in animals and plants by pairing to the mRNAs of protein-coding genes to direct their post-transcriptional repression (Bartel, 2009). *In vitro*, bacterial RNase III can be used to produce small noncoding RNA molecules as Dicer in eukaryotes, which is the essential enzyme to process miRNAs (Yang et al., 2002). It is suggested that they all have RIIID and dsRNA-binding domain that allows RNase III functions like Dicer, though more complex RIIID-containing proteins are found in Dicer (Bernstein et al., 2001; Calin-Jageman and Nicholson, 2003). Meanwhile, recent parallel studies have provided evidence for the high abundance of discrete microRNA-size small RNAs (msRNAs) in *S. mutans* and *Escherichia coli* (Lee and Hong, 2012; Kang et al., 2013). Overall, these findings suggest that msRNAs may participate in *rnc*-related regulation as post-transcriptional regulators in bacteria.

In this work, we identified the role of the *rnc* in exopolysaccharide synthesis, cariogenecity and its influence on the downstream *vicRKX* expression in *S. mutans* by mutant analyses and animal study. Furthermore, we detected the expressions of the candidate msRNAs targeting *vicRKX* by deep sequencing for analyzing the possible molecular mechanism. Here, we describe an additional level of control of *S. mutans* cariogenicity, msRNA-mediated post-transcriptional repression, which enriches the knowledge of virulence regulation in *S. mutans*. This paper provides an alternative avenue for further caries prevention research.

## Materials and methods

### Bacterial strains

The parent and *rnc* mutant strains of *S. mutans*, plasmid pDL278 and amplicons PcErm and aRnc used in this study are detailed in the Table S1. Synthetic oligonucleotides (Sangon Biotech, Shanghai, China) used and generated are listed in Table S2. The *rnc* gene sequence was obtained from the *S. mutans* UA159 genome dataset (http://www.ncbi.nlm.nih.gov/nuccore/AE014133.2) (Ajdic et al., 2002). To delete the *rnc* gene from *S. mutans* UA159, we used a ligation-PCR mutagenesis strategy, as previously described (Lau et al., 2002). The resulting *rnc* insertion–deletion mutant was named Smurnc. The *rnc* gene overexpression strain was constructed using the pDL278 shuttle vector with insertion of aRnc and was designated as Smurnc^+^. Ligation constructs and the shuttle vector were separately introduced into *S. mutans* UA159 using competence-simulating peptide (CSP)-induced natural transformation, and transformants that were independently resistant to erythromycin and spectinomycin were selected for recombination into the chromosome using PCR, followed by nucleotide sequence analysis. The level of *rnc* expression in the resulting mutants was monitored and compared to its level of expression in UA159 by quantitative real-time PCR (qRT-RCR) (Supplementary Material).

### Bacterial growth conditions

Unless stated otherwise, strains were grown in brain heart infusion (BHI) medium (Oxoid, Basingstoke, England) in an atmosphere of 80% N_2_ and 20% CO_2_ at 37°C. Appropriate antibiotics were added when culturing the mutant strains, i.e., erythromycin (10 μg/mL) for Smurnc, and spectinomycin (1200 μg/mL) for Smurnc^+^. Overnight cultures of UA159, Smurnc, and Smurnc^+^ were subcultured at 1:20 in fresh BHI for 2–3 h and the optical density (OD) at 600 nm (0.3) was then determined using an ultraviolet spectrophotometer system (Helsinki, Finland).

### Exopolysaccharide production assay

Modified biofilm growth was achieved as previously described (Yang et al., 2013). Each well of a 24-well plate, containing 2.0 ml sterile BHI with 1% sucrose, was inoculated with 0.5 ml of an exponential culture (OD_600nm_ = 0.3) of *S. mutans* and incubated anaerobically for 24 h. The established mature biofilms were used to measure the amounts of water-insoluble exopolysaccharides (WIGs) and water-soluble exopolysaccharides (WSGs) by anthrone–sulfuric acid colorimetric assay (Cury et al., 1997). All assays were performed in triplicate from at least three different experiments.

### Biofilm formation assays

Samples were obtained after 24 h of *in vitro* biofilm synthesis and from the *in vivo* SPF rat model. To determine the production and distribution of exopolysaccharides in the parent and *rnc* mutant *S. mutans* biofilms, we used scanning electron microscopy (SEM; FEI, Hillsboro, OR) (Li et al., 2001) and confocal laser scanning microscopy (CLSM; TSP SP2; Leica, Germany) based on *in situ* labeling of the exopolysaccharides with 1 μM Alexa Fluor 647 (Invitrogen, Eugene, OR, USA) and bacterial cells with 2.5 μM SYTO 9 (Invitrogen, Carlsbad, CA, USA) (Koo et al., 2010). The biofilms were washed three times with physiological saline and observed under confocal microscopy using Ar (514/488 nm) and He-Ne (543 nm) lasers. Under CLSM, the bacteria were stained green, while exopolysaccharides were stained red. A three-dimensional reconstruction of the biofilms from CLSM was analyzed using Imaris 7.0.0 software (Bitplane, Zurich, Switzerland). Three independent biofilm experiments were performed and images of three random fields of each group were captured.

### Animal study for detecting cariogenecity

All experiments involving rats were performed in accordance with the Chinese State Key Laboratory of Oral Diseases guidelines for animal welfare (NO.SCXK (111) 2009-09). Caries-susceptible, specific pathogen-free (SPF) Osborne-Mendel rats (IVC Experimental Animal Center of Public Health, Sichuan University, Chengdu, China) were used to investigate the *in vivo* effects of *rnc* mutations on the formation of dental caries. Four experimental groups were used: a blank was used as the negative control, UA159 was used as the positive control, Smurnc, and Smurnc^+^, where each group comprised 10 test animals. On days 23–28, each rat was infected orally once daily using 200 μl of a dense bacterial suspension that contained the UA159 strain, or the *rnc* deletion or expression mutants. The animals were sacrificed on day 50. The lower jaws were divided into right and left pieces and after three washes in phosphate-buffered saline, two pieces were selected for SEM and CLSM. The remaining section of the jaw was subjected to sonication using a Branson Sonifier 450 (Branson, Danbury, CT, USA) set for 10 s pulses at 70 W. The homogenized suspensions were stored at −20°C until further use. The lower jaws were dissected and immersed in fixative (10% buffered formalin phosphate) for a minimum of 72 h. The mandibular molars were sectioned and scored to determine fissure caries according to a modified Keyes system in this study. Detailed information about the modified Keyes system is provided in the Supplementary Material.

### Gene expression assay by qRT-PCR

qRT-PCR was performed to determine the transcript levels in samples of UA159 and *rnc* mutants *in vitro* and *in vivo*. RNA was extracted and purified using the classical TRIzol–chloroform protocol (Invitrogen, Carlsbad, CA). RNA purity (A260/A280) and concentration was assessed by a NanoDrop2000 spectrophotometer (Thermo Scientific, Waltham, MA, USA). Total RNA reverse transcription was performed with the RNeasy purification kit (QIAGEN, Valencia, CA, USA). qPCR was conducted as described by the manufacturer using a Bio-Rad CFX96 TM Real-time System (Bio-Rad, Hercules, CA, USA) and the Quantitect SYBR-Green PCR kit (QIAGEN, Valencia, CA, USA). qPCR was performed using specific primers for the *vicRKX* genes, and *gyrA* as a reference gene (Senadheera et al., 2005). All primers for RT-qPCR were obtained commercially (Sangon Biotech, Shanghai, China) and are listed in Table S2. Relative fold changes were determined using the 2^−Δ*ΔCT*^ method. We used technical replicates for each gene tested and we used at least three biological replicates in each experiment.

### Genatic analyses of *rnc* promoter structure

In the *S. mutans* UA159 dataset, we detected 108 intergenic base pairs between the *vicX* and *rnc* genes (gb|AE014133.2|:1443948–1444056). We first analyzed these intergenic noncoding sequences by FGENESB and BPROM programs for operon and promoter prediction, respectively (http://linux1.softberry.com/berry.phtml) (Solovyev and Tatarinova, 2011). Co-transcription assay were conducted with templates of DNA and reverse transcribed cDNA extracted from UA159. Genomic DNA was extracted and purified by using QIAamp DNA micro Kit (QIAGEN Sciences, MD, USA) and cDNA was conducted as previously described. qPCR was performed by using the specific primes for this assay (Table S2), and *rnc* as a reference gene.

### Identification of msRNAs by deep sequencing

Samples of UA159, Smurnc, and Smurnc^+^ were grown in fresh BHI until mid-exponential phase. Total RNA was isolated as described above. Briefly, cDNA libraries were generated according to the Illumina TruSeq™ SmallRNA sample preparation protocol (online Figure S2). Size-fractionated RNA fragments of ca 18–150 nt in length were isolated using gel extraction and ethanol precipitation. After ligation with a pair of adaptors 5′ (5′-GUUCAGAGUUCUACAGUCCGACGAUC-3′) and 3′ (5′-UGGAAUUCUCGGGUGCCAAGG-3′), small RNA molecules were subjected to reverse transcription-PCR to obtain single-stranded cDNAs and then further amplified by PCR. After purification, the PCR products were used for sequencing by Illumina technology on HiSeq™ 2000 (Illumina, San Diego, CA, USA) by BGI, Shenzhen China.

### Bioinformatic analysis for selecting msRNAs

Raw sequences were processed using Illumina PIPELINE software and then subjected to a series of data filtration steps for analyses. After filtering out low quality reads and trimming the adaptor sequences, high quality clean sequences mapping to intergenic region (IGR) and antisense mRNA (AM) were selected. The annotated sequences were further screened to remove rRNA and tRNA by searching against the NCBI Genbank database (http://www.ncbi.nlm.nih.gov/nuccore/AE014133) and the Rfam database (http://www.sanger.ac.uk/software/Rfam). RNAfold software (http://rna.tbi.univie.ac.at/cgi-bin/RNAfold.cgi) was employed to predict msRNA candidates by exploring the secondary structure and the minimum free energy. Sequences and structures of the putative msRNAs satisfied the criteria of forming hairpin miRNAs, and secondary structures of hairpins have free energy of hybridization ≤ 0 kcal/mol. Differential-expression analysis was performed using DEseq (Anders and Huber, 2010). Differential expression of msRNAs was performed based on the reads from the UA159, Smurnc, and Smurnc^+^ libraries. Fold-change was calculated according the following equation: log_2_ Ratio = log_2_ (Smurnc reads/UA159 reads). *P*-value was calculated as previously described (Audic and Claverie, 1997). If the standard expression of a given msRNA was zero, its expression value was modified to 0.01. Significant difference in msRNA expression was assigned to sequences with a *P* < 0.05 and |log_2_Ratio| ≥ 6.64. Target gene predictions were obtained from IntaRNA (http://rna.informatik.uni-freiburg.de:8080/IntaRNA.jsp). GO enrichment analysis of target gene candidates was carried out using the GO terms in the database (http://www.geneontology.org/) and we set the candidates as *vicRKX*. Mis-matching analyses were conducted based the base-pairing between candidate msRNAs and target gene mRNA.

### Stem-loop qRT-PCR for verifying msRNA expression

Stem-loop qRT-PCR with SYBR Green was performed to verify the expression patterns revealed by RNA-seq (Chen et al., 2005). Total RNA was isolated from UA159 and *rnc* mutant strains as described above, and first strand cDNA was synthesized using specific stem-loop primers listed in Table S3. The reverse transcripts were used with the RevertAid First Strand cDNA Synthesis kit (RevertAid, Thermo scientific) following the manufacturer's protocol. qPCR was performed using SYBR® Green Realtime PCR Master Mix (QIAGEN, Valencia, CA, USA) and carried out in a Bio-Rad CFX96 TM Real-time System (Bio-Rad, Hercules, CA, USA). The reaction conditions were 95°C for 2 min, followed by 45 cycles of 95°C for 15 s, 45–50°C (according to the Tm of each pair of primers) for 40 s, 60°C for 30 s, and then fluorescence levels were measured at 60°C. The expression level of each msRNA gene was determined based on three replicates.

### Statistical analyses

Statistical analyses of the data were performed using SPSS 16.0 (SPSS Inc., Chicago, IL, U.S.). The Shapiro–Wilk test and Bartlett's test were first used to assess whether the data were parametric or not. For parametric testing, Fisher's tests and one-way ANOVA were used to detect the significant effects of variables. For nonparametric testing, the Kruskal–Wallis test and least significant difference (LSD) multiple comparisons were used. The differences of the means of data were considered significant if the *P* < 0.05.

## Results

### *rnc* promotes exopolysaccharide synthesis and alters biofilm morphology

**Figure 1:**
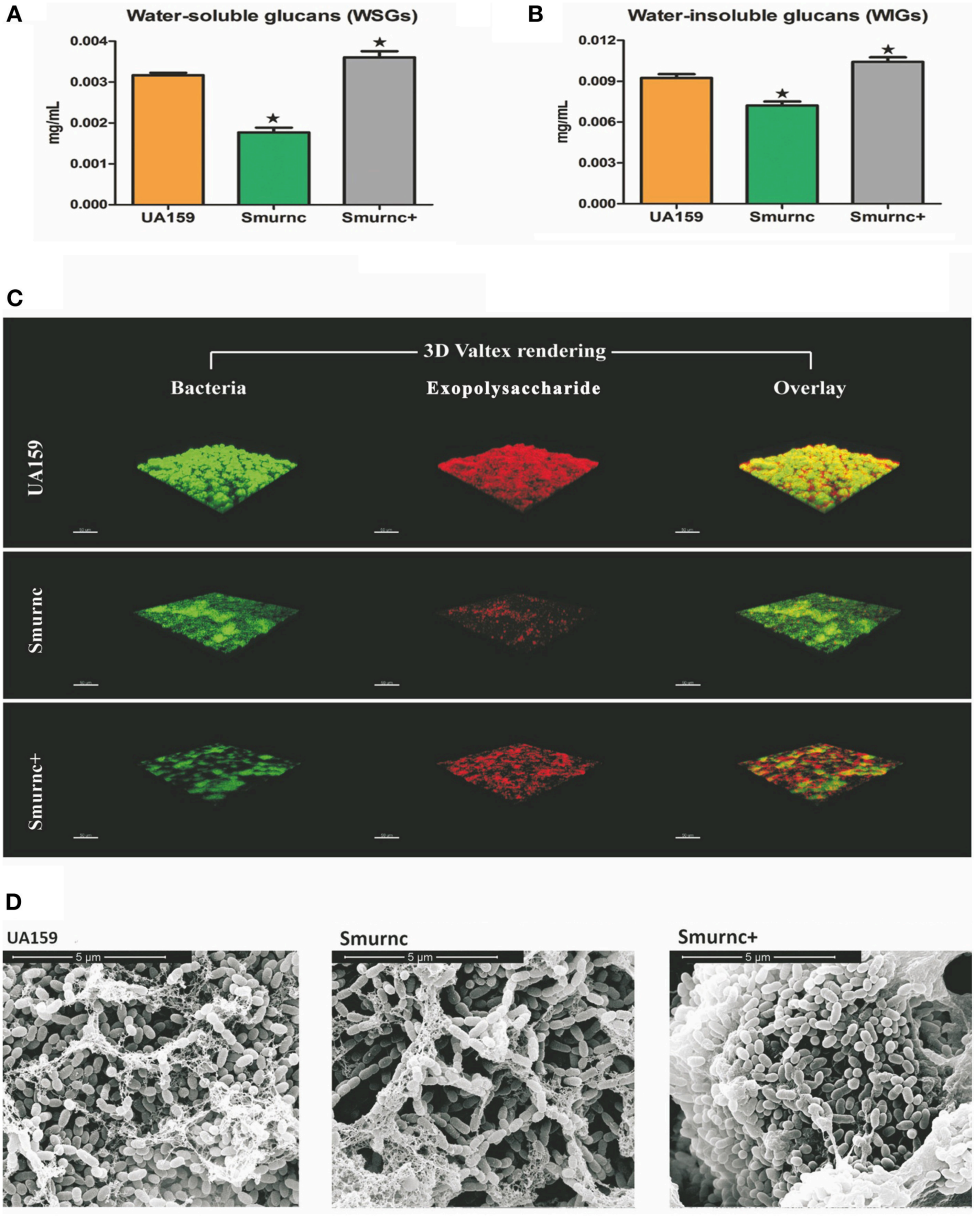
**The effects of *rnc* gene on exopolysaccharide synthesis and biofilm morphology in *S. mutans***. The anthrone assay was used to determine glucan productions in UA159, Smurnc, and Smurnc^+^ biofilms at 24 h. WSGs **(A)** and WIGs **(B)** were both calculated. The Smurnc strain decreased the amount of glucans, whereas the Smurnc^+^ strain increased the amount. The error bars represent standard deviation values. Asterisks indicate significant differences (*P* < 0.05). **(C)** Representative three-dimensional images of production and distribution of exopolysaccharide matrix in the biofilm architecture. Mature biofilms (24 h) formed by *S. mutans* UA159 and *rnc* mutant strains in the presence of 1% (wt/vol) sucrose by CLSM. Images were taken at 63× magnification. **(D)** SEM observation of the architecture of *S. mutans* mature biofilms (24 h). Images of biofilm structures formed by UA159 and *rnc* mutant strains were taken at 20,000× magnification.

To explore the biological function of *rnc*, we successfully constructed an *rnc* deletion mutant Smurnc and *rnc* overexpression mutant Smurnc^+^. We found Smurnc and Smurnc^+^ possessed similar growth rates compared to UA159 in planktonic and biofilm forms (Figure S1). Detected by the anthrone assay, it showed that both WSGs and WIGs decreased significantly in Smurnc but increased in Smurnc^+^ (*P* < 0.05, Figures 1A,B). However, the real production of WIGs in Smurnc^+^ (0.0036 ± 0.0001 mg/mL) was slightly elevated comparing to UA159 (0.0032 ± 0.0001 mg/mL). CLSM was used to observe the biofilms in three-dimensions in both parent UA159 or *rnc* mutant strains (Figure 1C). In UA159, the exopolysaccharides and bacteria formed a compact lattice-like structure with large “mushroom-like” clumps of colonies and conspicuous channels. In contrast, Smurnc exhibited a reduced capacity to synthesize an exopolysaccharides matrix similar with anthrone assay. For Smurnc^+^, exopolysaccharides was slightly less than UA159. The microcolonies of the *rnc* mutants, including Smurnc and Smurnc^+^, were decreased in terms of both size and number, and their biofilms lost their typical branch-like structure. The decreased bacteria colony may be caused by the abnormal cell arrangement shown by SEM (Figure 1D). According to the results of SEM, in Smurnc^+^ the bacteria preferred to gather into dense clusters covered by floating exopolysaccharides and in Smurnc bacteria appeared to scatter to form larger tunnels among the clumps. The biofilm of them both were less condensed than the biofilm in UA159.

### *rnc* affects the cariogenicity of *S. mutans* in a SPF rat model

We next examined the role of *rnc* in the pathology of dental caries in a SPF rat model. Parent and *rnc* mutant strains were inoculated in the rat oral cavity. The lower dentition in rats was used to investigate the effects of the *rnc* gene on cariogenic characteristics. We verified that a knockout of *rnc* reduced the cariogenicity of *S. mutans* (Table 1). Smurnc and Smurnc^+^ exhibited a significantly decreased number of sulcal caries compared with those in the UA159 group after three weeks *in vivo* (*P* < 0.05, E). Interestingly, the severity of limited sulcal caries, designated as slight dentinal (Ds), was increased in the Smurnc groups, while the Smurnc^+^ mutants did not show any differences in comparison to UA159.

**Table 1:** **Sulcal caries unit scores in different groups**.

**Group**	**Incidence of sulcal caries**			**Severity of sulcal caries**			
	**E**^a^			**Ds**^a^		**Dx**^a^	
Control	F^b^	18	32^*^	3	6^*^	0	0	
	M^b^	14		3		0	
UA159	F	30	60	15	24	3	3
	M	30		9		0	
Smurnc	F	24	48^*^	18	42^*^	9	12
	M	24		24		3	
Smurnc+	F	21	45^*^	9	24	3	9
	M	24		15		6	

a *The linear extent of lesions in point plane and recording the depth of penetration are under 3 headings: enamel only (E), slight dentinal (Ds) and extensive dentinal (Dx)*.

b *Each of 4 groups was divided into female (F) and male (M) two sub-groups*.

*The data represent the sulcal carious lesions score according to a modified Keyes in this study. Shapiro–Wilk tests and Bartlett's tests showed that the data were nonparametric. By using UA159 as a reference, significant differences were determined using the Kruskal–Wallis test and least significant difference (LSD) multiple comparisons test. Asterisks indicate P < 0.05*.

**Figure 2:**
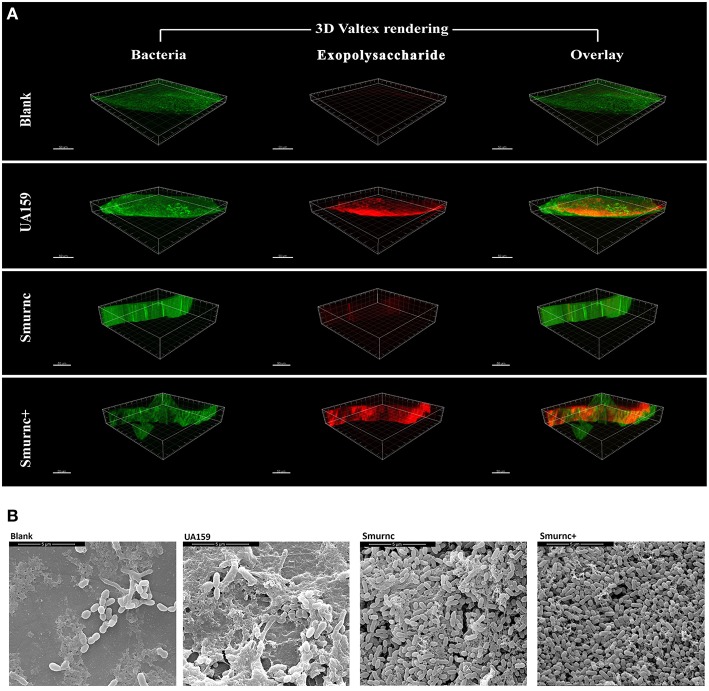
**The influences of *rnc* gene on *S. mutans* cariogenicity in the SPF rat model. (A)** Representative three-dimensional images of biofilms that formed on occlusal plane of rat mandibular molars in four different groups by CLSM. Images were taken at 63× magnification. **(B)** SEM observation of the architecture of biofilms that formed on occlusal plane of rat mandibular molars. Images were taken at 20,000× magnification.

Furthermore, the *rnc* mutants had changes in exopolysaccharide production in the *in vivo* study. In a representative three-dimensional view obtained by CLSM, the mutants exhibited a similar inclination toward exopolysaccharide synthesis compared with that observed *in vitro*. In particular, compared to the parent strain UA159, Smurnc exhibited a significantly decreased level of exopolysaccharides whereas Smurnc^+^ showed a similar level of exopolysaccharide production (Figure 2A). To obtain a better view of the exopolysaccharide distribution in the biofilms, SEM images of rat molars were also captured. At 20,000 × magnification, we observed that the exopolysaccharides produced by the *rnc* mutants was much sparser and thinner than that produced by UA159 (Figure 2B).

### *rnc* represses *vicRKX* expression *in vitro* and *in vivo*

**Figure 3:**
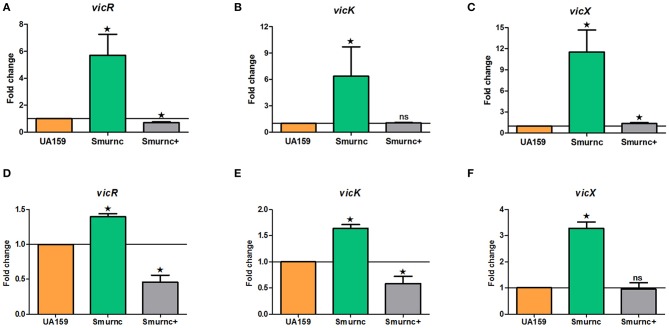
**Dynamics of the expression of *vicR/K/X* genes in interaction with *rnc* gene among three types of strains. (A–C)** Relative expression levels of *vicR/K/X* were calculated during the mid-exponential phase *in vitro*. **(D–F)** Relative expression levels of *vicR/K/X* were calculated in SPF rat model *in vivo*. The fold changes were shown after standardization relative to *gyrA* using UA159 as a reference. The data were represented as the means and standard deviation values. Asterisks indicate significant differences (*P* < 0.05); ns, no significant difference.

Our previous studies identified that *rnc* was involved in exopolysaccharide synthesis, biofilm formation and cariogenicity in *S. mutans*. We analyzed the expressions of several exopolysaccheride synthesis-related genes in Smurnc and found these genes all up-regulated (Figure S3). For exploring the promising repression mechanism of *rnc*, we first analyzed the specific expression of *vicRKX* at the transcriptional level in parent UA159 and *rnc* mutant strains both *in vitro* biofilm study and *in vivo* animal study. The trend of *vicRKX* expressions was similar both *in vitro* and *in vivo*. Specifically, when compared to parent strain UA159, the transcription levels of all of the *vicR/K/X* genes increased remarkably in Smurnc (*P* < 0.05, Figure 3), whereas *vicR* transcription was reduced in Smurnc^+^ (*P* < 0.05, Figures 3A,D) and *vicK/X* transcription appeared stable level. Based on these results, we further investigated the molecular mechanism underlying this peculiar phenomenon.

### Genetic analysis indicates *rnc* is not a part of *vicRKX* tricistronic operon

Knowledge of the co-transcription relationship between a regulator gene and structural genes is helpful to illustrate its potential regulation mechanism. Thus, we investigated the transcriptional relationship between *rnc* and the *vic* locus.

**Figure 4:**
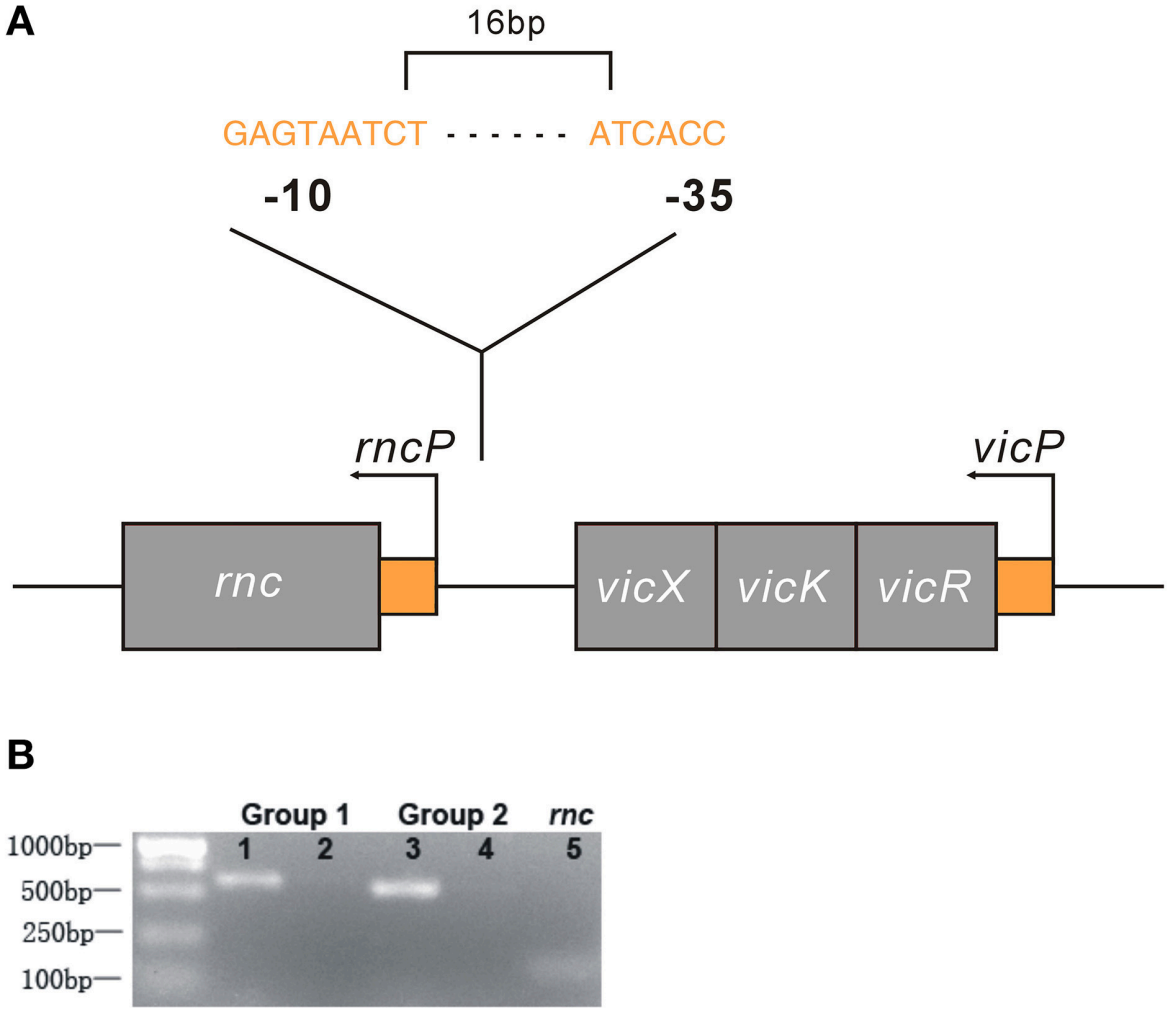
**Genetic analyses of the *rnc* and *vicRKX* gene loci. (A)** Predicted genetic structure of *rnc* and *vicRKX* loci by FGENESB and BPROM. The fragment shown was from the *S. mutans* chromosome, which contained the intact *rnc, vicX, vicK*, and *vicR* genes, shown from left to right. The entire sequence was known for this region. Arrows indicated the initiation sites for the *vicP* and *rncP* promoters. The supposed sequences of -10 and -35 boxes in *rncP* were GAGTAATCT and ATCACC, respectively. **(B)** PCR of *rnc* and *vicX* co-transcription assays. Two sets of specific PCR primers were used for the co-transcription assay. Lanes 1 and 3 were used for UA159 DNA, and lanes 2 and 4 were used for UA159 cDNA, which was reverse transcribed from RNA. Line 5 was used as a reference to verify the feasibility of the cDNA.

We analyzed the NCBI database and found that *rnc* and the *vicRKX* tricistronic operon were transcribed in the same direction and that they were separated by 108 bp of intergenic noncoding DNA. In most organisms, knowledge of operon structure is based on computational methods. We first used FGENESB to predict and it showed that these genes were not located in one operon. Then, we employed BPROM to analyze the existence of a promoter within the 108 intergenic base pairs. Similar to FGENESB, it identified a promoter that could initiate *rnc* transcription. The predicted genetic structure of *rnc* and the *vicRKX* locus are shown in Figure 4A. Furthermore, the co-transcription assay directly demonstrated that *rnc* and *vicX* were not transcribed as a single mRNA (Figure 4B). The results of these experiments showed that *rnc* and *vicRKX* were not located in the same operon and that they are expressed separately.

### *rnc* represses *vicRKX* expressions at the post-transcriptional level via msRNAs

**Figure 5:**
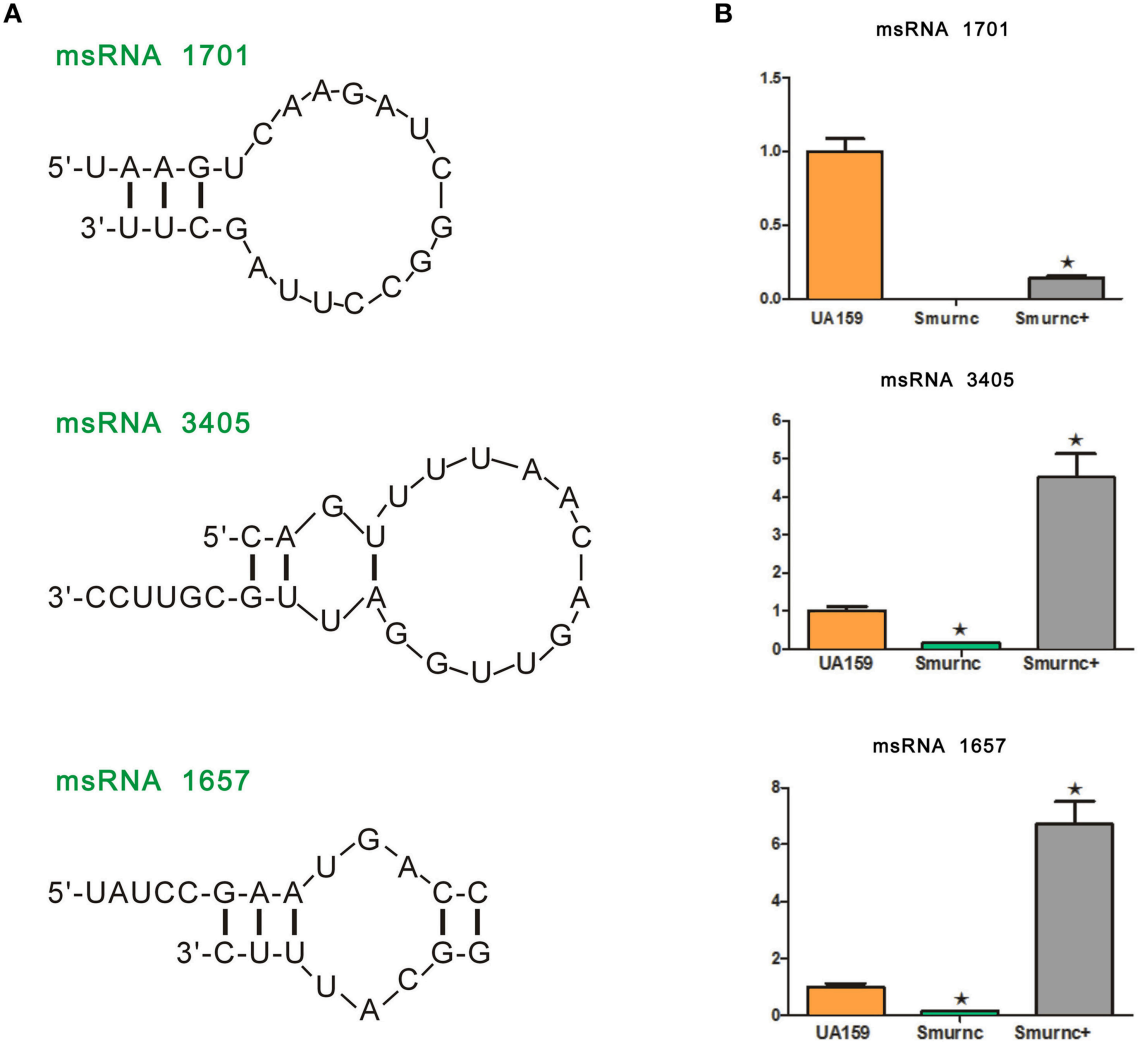
**Presentation and validation of the deep sequencing data by secondary structure prediction and expression analyses. (A)** Predicted secondary RNA structures formed by three validated msRNAs, registered by deep sequencing. **(B)** Comparison of expression levels of three validated msRNAs in UA159 and *rnc* mutations by stem loop RT-PCR. The fold changes were shown after standardization relative to 16s RNA using UA159 as a reference. The data were represented as the means and standard deviation values. Asterisks indicate significant differences (*P* < 0.05); ns, no significant difference.

Deep sequencing allows the identification and quantification of small noncoding RNA molecules. Using this technique, we analyzed msRNAs targeting *vicRKX* in UA159 and the *rnc* mutants. The detailed filtering steps are shown in Figure S4. Briefly, we removed low-quality reads and chose clean reads in cultures of UA159 and the *rnc* mutants. Taking several bioinformatic analyses into consideration, we finally extracted three putative molecules as validated msRNAs, which correspond to msRNA 1701, 3405, and 1657 as detailed in Table 2. The ability of miRNAs to fold back on themselves to form distinctive hairpin structures is known to be a critical factor that differentiates these from other classes of small RNAs (Bartel, 2009). Thus, we predicted the potential secondary structures of these msRNAs (Figure 5A), which showed they all could form hairpins structures.

**Table 2:** **The detailed information of three validated msRNAs in *S. mutans***.

**Id-ncRNA**	**Id-mRNA**	**Gene length [nt]**	**Energy [kcal/mol]**	**Position -mRNA**	**log_2_Ratio (Smurnc/UA159)**	**Mis-matching**	**Interaction-mRNA(5′–3′)**	**Sequence**
msRNA 1701	*vicK*	23	−5.66014	182–204	−14.50724764	0	AGGUUGAGGAUAACUCUGACUUG	TAAGTCAAGATCGGCCTTAGCTT
msRNA 3405	*vicR*	26	−11.7443	583–610	−14.01192235	0	GGAGAUGUCCGUACUGUUGAUGUUACUG	CAGTTTTAACAGTTGGATTGCGTTCC
msRNA 1657	*vicK*	24	−7.9363	405–433	−15.19147178	0	GGAGUGCAAUAUUCUGGAUAUUUUGGAUG	TATCCGAATGACCGGCGGCATTTC

Bioinformatic analysis predicted the presence of these particular msRNAs and stem-loop qRT-PCR was applied to validate the deep sequencing data. Expression levels of the three selected msRNAs were calculated using qPCR, and it revealed a rough correlation between the number of msRNAs and their cellular level. More importantly, expression of the three msRNAs corresponded to that seen with deep sequencing, suggesting that deep sequencing data were reliable. We found that there were significant differences in the expression of selected msRNAs between UA159 and the *rnc* mutants (*P* < 0.05, Figure 5B). That is, compared with the UA159 strain, the expression of msRNA 1701, 3405, and 1657 in the Smurnc was significantly down-regulated by 0.01-, 0.15- and 0.13-fold, respectively. Meanwhile, we found their expression levels were negatively correlated with *vicRKX* but positively correlated with *rnc* by combining mRNA and msRNA qPCR analyses, indicating *rnc* probably regulates *vicRKX* expression through msRNAs by post-transcriptional repression.

## Discussion

The *rnc* gene has long been considered as a regulator that is involved in bacterial physiology. Previous investigations showed in *Streptomyces coelicolor rnc* was essential for the production of antibiotics actinorhodin and undecylprodigiosin (Sello and Buttner, 2008; Xu et al., 2010). In *Streptomyces antibioticus*, it has been suggested as a global regulator of actinomycin production (Lee et al., 2013). In the present study, we found that the expression of *rnc* was positively correlated with exopolysaccharide synthesis. Here, we employed the anthrone assay to detect the production of WSGs and WIGs. Comparing to UA159, the amounts of WSGs and WIGs in Smurnc was significantly declined, which could be used to explain the less condensed biofilm structure. It is suggested that reductions in the exopolysaccharide matrix might have resulted in weakened connections among microcolonies and, therefore, the presence of more scattered microcolonies. The disorder of biofilm formation, which was mainly caused by altered exopolysaccharide production, could be vividly detected by CLSM and SEM *in vitro* and *in vivo*. Similar with our study, exopolysaccharide is considered to provide mechanical integrity/stability for biofilm formation, and provide supporting frame for continuous growth of the microcolonies (Koo et al., 2009, 2013). Interestingly, the production of exopolysaccharides was not representative in Smurnc^+^ (Figures 1C, 2A). In other words, the Smurnc^+^ strain exhibited different amounts of exopolysaccharide matrix in different conditions. It could be speculated that the introduction of an exogenous plasmid vector carrying *rnc* gene may interfere with the role of the whole genome in regulating intracellular homeostasis in *S. mutans*. All the available evidences showed that exopolysaccharide is a critical virulence factor of dental caries (Bowen, 2016). It provides an abundance of primary binding sites and forms the core of the matrix-scaffold in cariogenic biofilms (Nobbs et al., 2009; Bowen and Koo, 2011; Xiao et al., 2012). Collectively, our analysis of the *rnc* demonstrate that *rnc* has a potential biological function where by it can regulate exopolysaccharide synthesis and further alter the biofilm morphology and the cariogenicity of *S. mutans*. Therefore, we propose that *rnc* is a new anti-virulence gene locus and it will provide important insights into the genetic pathways that control exopolysaccharide synthesis in *S. mutans*.

Herein, we firstly studied the expression of several exopolysaccharide synthesis-related genes in Smurnc. However, the results showed that all these genes were typically repressed by *rnc* (Figure S3). It indicated that the regulation of *rnc* on exopolysaccharide synthesis was conducted by a genetic network involving not only formation-related but also disintegration-related genes. Interestingly, the results of this study showed that the expressions of *vicRKX* and *gtfB* both were markedly increased compared to UA159, which was consistent with VicR being a positive regulator of the *gtf* genes of previous studies (Senadheera et al., 2005, 2007). The *rnc*-mediated regulation of exopolysaccharides needs more exploration and our further research will focus on this proposal. TCS is one of the most prevalent means to mediate the response of bacteria to a wide range of signals and stimuli (Laub and Goulian, 2007). Among these genes, the *vic* locus has been reported to encode VicRK TCS and it underscores its tremendous versatility and utility to *S. mutans* (Senadheera et al., 2009, 2012; Ayala et al., 2014). Indeed, the localization of *vicRKX* and *rnc* is conjoint in *S. mutans* genome. Thus, we chose the *vicRKX* genes for an in-depth study of the possible regulatory mechanism of *rnc*-mediated repression.

Determining whether the regulatory effects of *rnc* on *vicRKX* are limited to one operon would help us better understand *rnc*-mediated virulence expression in *S. mutans* genome. Here, all the genetic location analyses showed that *rnc* was not a part of *vicRKX* tricistronic operon. Each operon is a series of genes transcribed in a single mRNA, often identified by the presence of promoters and terminators. In bacteria, regulator genes can be located within an operon, adjacent to it, or far away from it. It has been suggested that bacterial genes with similar functions may be located near each other or even in a single operon, which are major structural and regulatory features of prokaryotic genomes (Okuda et al., 2007). However, there are several global regulators that can regulate distal genes and these seem to have more powerful effects (Bratlie et al., 2010). Considering the global regulation of *rnc* in *S. antibioticus, rnc*-mediated regulation in *S. mutans* appears to operate in a similar fashion (Lee et al., 2013).

For the first time, we identified three putative msRNAs that target *vicRKX* in *S. mutans*, and their expression levels were negatively correlated with *vicRKX* but positively correlated with *rnc*. In *S. mutans*, it appears that *rnc* participates in the post-transcriptional regulation of *vicRKX*. The *rnc* gene is believed to encode RNase III in *S. mutans* (Fonfara et al., 2014). Members of the RNase III family include bacterial RNase III and the eukaryotic proteins Drosha and Dicer (Court et al., 2013). All of these proteins have similar core domains, RIIID and dsRBD (Calin-Jageman and Nicholson, 2003). Drosha and Dicer are critical in miRNA-related processes. In eukaryotes, Drosha catalyzes the initial processing of the stem loop (hairpins) of primary miRNAs (pri-miRNAs) into short, hairpin, precursor miRNAs (pre-miRNAs), and Dicer further processes pre-miRNAs into miRNAs, which regulate more than half of all mammalian coding sequences via RNA interference (RNAi) (Tomari and Zamore, 2005; Friedman et al., 2009). A recent study reported a novel RNA cleavage event in eukaryotes and it was suggested that some of the miRNAs generated by Dicer might be obtained via this cleavage process, which is the same as that mediated by RNase III in *E. coli* (Gu et al., 2012). The existence of msRNAs in *E. coli* has been predicted and verified (Kang et al., 2013). This newly identified cleavage event may therefore represent a possible mechanism for msRNA production in bacteria. Here three msRNAs were predicted to target the downstream *vicRKX* and their expression levels were detected. The results showed that the expression of *rnc* directly and positively affected the levels of the three msRNAs. The evidence here implies that *rnc* regulates msRNA expression or is involved in their synthesis. Further investigation of the formation of msRNAs and the specific functions of msRNA-related mechanisms should provide new insights into post-transcriptional regulation in prokaryotes and, more importantly, their evolution.

## Author contributions

TH and X-RM designed research; M-YM, Y-MY, K-ZL, LL, ML, YY, J-XY, and RZ acquired the data; M-YM and Y-MY analyzed and interpreted the data; and M-YM wrote the main manuscript text. All authors discussed the results and commented on the manuscript.

### Conflict of interest statement

The authors declare that the research was conducted in the absence of any commercial or financial relationships that could be construed as a potential conflict of interest.

## Supplementary material

Table S1 **Bacterial strains, plasmid, and amplicons used in this study**.

Table S2 **List of oligonucleotide primers used in this study**.

Table S3 **Sequences of primers used for stem-loop qRT-PCR analysis**.

Figure S1 **Growth curves of *S. mutans* UA159, Smurnc, and Smurnc+**. Each datum point is the average of nine independent OD values per sample. The results shown are representative of three independent experiments conducted with the mutants and UA159 parent strains.

Figure S2 **Workflow to generate cDNA libraries**. The directions of the arrow marks denote the flow of total RNA processing and sequential.

Figure S3 ***rnc* affected the expression of several exopolysaccheride- related genes at the transcriptional level**. The relative levels of target gene expression in UA159 and Smurnc were determined at mid-exponential phase *in vitro*. The fold changes are shown after standardization relative to *gyrA* using UA159 as a reference.

Figure S4 **Workflow to analyze and select the validated msRNAs**. The directions of the arrows denote the flow of data processing and sequences. The shapes of the boxes have no particular significance, while the descriptions within the boxes represent the steps corresponding to data filtering.
