# Supplementary material for: The rnc Gene Promotes Exopolysaccharide Synthesis and Represses the vicRKX Gene Expressions via MicroRNA-Size Small RNAs in Streptococcus mutans
Source: Front Microbiol. 2016 May 10;7:687. doi: 10.3389/fmicb.2016.00687 (PMC4861726; doi:10.3389/fmicb.2016.00687)
Supplement: Table S1 — Bacterial strains, plasmid, and amplicons used in this study. [file Table1.DOCX]

| **Strains, plasmid, amplicon** | **Relevant characteristic(s)** | **Source or reference** |
| --- | --- | --- |
| ***S.mutans* strains** |  |  |
| UA159 | Wild type | ATCC 700610 |
| Smurnc | UA159 *rnc::ermAM* Em^H^ | This study |
| *Smurnc+* | UA159 derived; *rnc* overexpression:Sp^H^ | This study |
| **Plasmid** |  |  |
| pDL278 | *E. coli*-streptococcal shuttle vector; Sp^H^ | ([Wen et al., 2011](#_ENREF_1)) |
| ***Amplicons*** |  |  |
| *PcErm* | Erm marker amplified using *ermAM* cassette | This study |
| *aRnc* | *rnc*-5’::PcErm:*:rnc*-3’fragment used for  allelic replacement of *rnc* gene | This study |

**TABLE S1 Bacterial strains, plasmid, and amplicons used in this study**

^H.^ Em, erythromycin; Sp, spectinomycin.

Wen, Z.T., Nguyen, A.H., Bitoun, J.P., Abranches, J., Baker, H.V., and Burne, R.A. (2011). Transcriptome analysis of LuxS-deficient Streptococcus mutans grown in biofilms. *Mol Oral Microbiol* 26**,** 2-18. doi: 10.1111/j.2041-1014.2010.00581.x.
