# Supplementary material for: The rnc Gene Promotes Exopolysaccharide Synthesis and Represses the vicRKX Gene Expressions via MicroRNA-Size Small RNAs in Streptococcus mutans
Source: Front Microbiol. 2016 May 10;7:687. doi: 10.3389/fmicb.2016.00687 (PMC4861726; doi:10.3389/fmicb.2016.00687)
Supplement: Table S2 — List of oligonucleotide primers used in this study. [file Table2.DOCX]

| **Primers** | **Nucleotide sequences** | **Annealing temp (°C)** | **size (bp)** |
| --- | --- | --- | --- |
| **PCR-ligation mutagenesis^a^** | | | |
| rnc-P1 | 5’ACCATCACTTTTGGGGATATTG 3’ | 51.3 | 697 |
| rnc-P2 | 5’GGCCGGCCGTTTAGGAGGCGATGCTCATTA 3’ |  |  |
| rnc-P3 | 5’**GGCGCGCC**CAGAGCAAGAGGCTGCTAAAA 3’ | 52.5 | 621 |
| rnc-P4 | 5’CGATCCAAATTATCCTGAGTCTG 3’ |  |  |
| erm-PF | 5’ **GGCGCGCC**CCGGGCCCAAAATTTGTTTGAT 3’ | 52.3 | 876 |
| erm-PR | 5’ GGCCGGCCAGTCGGCAGCGACTCATAGAAT 3’ |  |  |
| **Cloning^b^** | | | |
| rnc+pro-F | 5’GAC**GGATCC**TGGACAGCTTAAAAGGAAGAATG 3’ | 51.2 | 150 |
| rnc+pro-R | 5’ GACGAATTCTTAAGAACCTCGTTGAAGTTTTT 3’ |  |  |
| **qRT-PCR^c^** | | | |
| vicR-F | 5’ CGCAGTGGCTGAGGAAAATG 3’ | 60 | 157 |
| vicR-R | 5’ ACCTGTGTGTGTCGCTAAGTGATG 3’ |  |  |
| vicK-F | 5’ CACTTTACGCATTCGTTTTGCC 3’ |  | 102 |
| vicK-R | 5’ CGTTCTTCTTTTTCCTGTTCGGTC 3’ |  |  |
| vicX-F | 5’ TGCTCAACCACAGTTTTACCG 3’ |  | 127 |
| vicX-R | 5’ GGACTCAATCAGATAACCATCAGC 3’ |  |  |
| rnc-F | 5’ CAGCCTCTTGCTCTGCTAATTTT 3’ |  | 150 |
| rnc-R | 5’ AAGTTGACGGGGATGTTTTGAT 3’ |  |  |
| gyrA-F | 5’ ATTGTTGCTCGGGCTCTTCCAG 3’ |  | 105 |
| gyrA-R | 5’ ATGCGGCTTGTCAGGAGTAACC 3’ |  |  |
| **PCR****^d^** | | | |
| rnc-vicX-F1 | 5’ CGAACAATCATCGAACGCAT 3’ | 51.1 | 770 |
| rnc-vicX-R1 | 5' CAACCACAGTTTTACCGCCT 3' |  |  |
| rnc-vicX-F2 | 5’ CGAACAATCATCGAACGCAT 3’ | 49.5 | 439 |
| rnc-vicX-R2 | 5’ CGAACAATCATCGAACGCAT 3’ |  |  |

**TABLE S2. List of oligonucleotide primers used in this study**

1. AscI restriction sites are in boldface, and FseI restriction sites are underlined.
2. BamHI restriction sites are in boldface, and EcoRI restriction sites are underlined.
3. qRT-PCR, quantitative reverse transcription–polymerase chain reaction.
4. Primers for PCR of *rnc* and *vicX* co-transcription assay.
