# Supplementary material for: The rnc Gene Promotes Exopolysaccharide Synthesis and Represses the vicRKX Gene Expressions via MicroRNA-Size Small RNAs in Streptococcus mutans
Source: Front Microbiol. 2016 May 10;7:687. doi: 10.3389/fmicb.2016.00687 (PMC4861726; doi:10.3389/fmicb.2016.00687)
Supplement: Table S3 — Sequences of primers used for stem-loop qRT-PCR analysis. [file Table3.DOCX]

**Table S3 Sequences of primers used for stem-loop qRT-PCR analysis**

| **msRNA** | | **Reverse transcription** | **qPCR-primer** | |
| --- | --- | --- | --- | --- |
| **ID** | **sequence** | **RT-primer** | **Primer F** | **Primer R** |
| msRNA 1701 | TAAGTCAAGATCGGCCTTAGCTT | CCTGGCAGTGATGTTGCGGTCTGCCAGGATCGAA | TAAGTCAAGATCGGCCTTA | CAGTGATGTTGCGGTCT |
| msRNA 3405 | CAGTTTTAACAGTTGGATTGCGTTCC | CCTGGCAGTGATGTTGCGGTCTGCCAGGGCAAGG | CAGTTTTAACAGTTGGATTG | CAGTGATGTTGCGGTCT |
| msRNA 1657 | TATCCGAATGACCGGCGGCATTTC | CCTGGCAGTGATGTTGCGGTCTGCCAGGTGTAAAG | TATCCGAATGACCGGCGGCA | CAGTGATGTTGCGGTCT |
