# Supplementary figures and images for: The rnc Gene Promotes Exopolysaccharide Synthesis and Represses the vicRKX Gene Expressions via MicroRNA-Size Small RNAs in Streptococcus mutans
Source: Front Microbiol. 2016 May 10;7:687. doi: 10.3389/fmicb.2016.00687 (PMC4861726; doi:10.3389/fmicb.2016.00687)

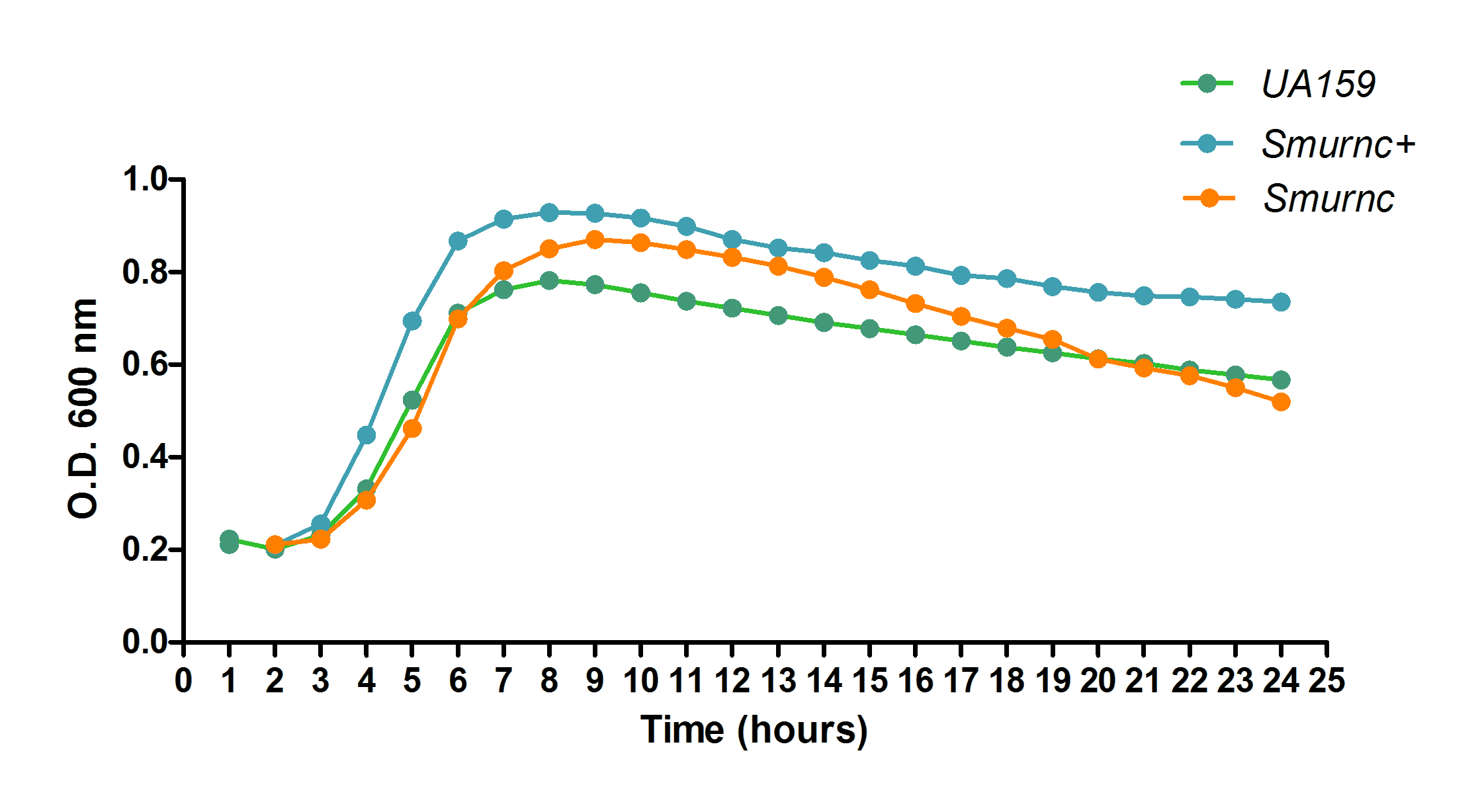

Supplement: Figure S1 — Growth curves of S. mutans UA159, Smurnc, and Smurnc+. Each datum point is the average of nine independent OD values per sample. The results shown are representative of three independent experiments conducted with the mutants and UA159 parent strains. [file Image1.TIF]

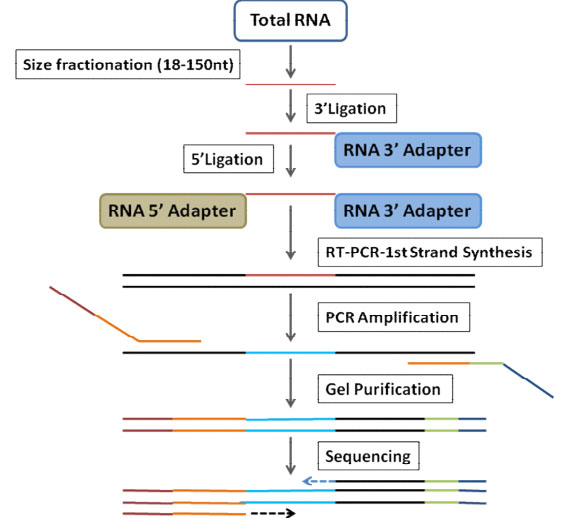

Supplement: Figure S2 — Workflow to generate cDNA libraries. The directions of the arrow marks denote the flow of total RNA processing and sequential. [file Image2.JPEG]

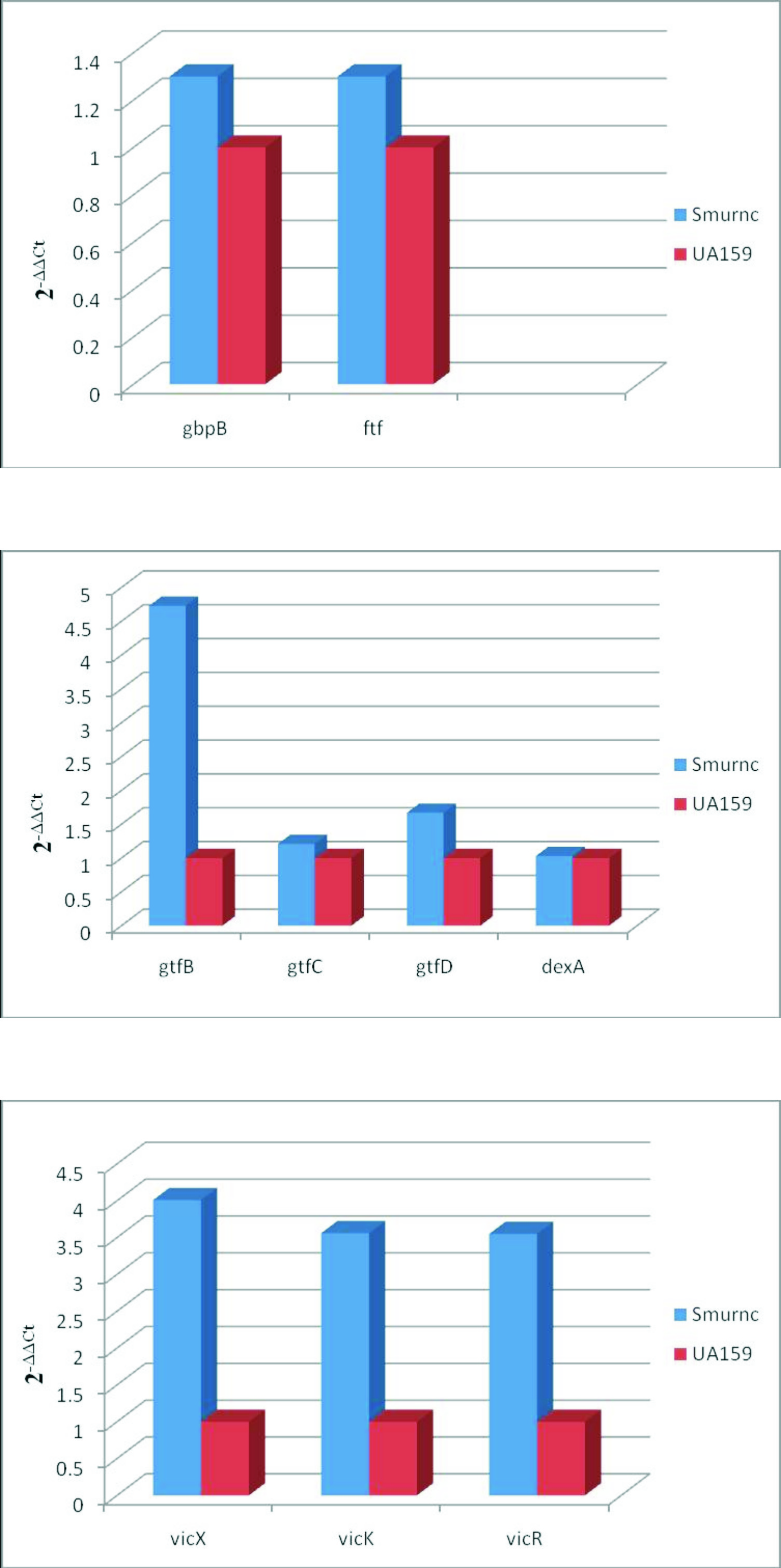

Supplement: Figure S3 — rnc affected the expression of several exopolysaccheride- related genes at the transcriptional level. The relative levels of target gene expression in UA159 and Smurnc were determined at mid-exponential phase in vitro. The fold changes are shown after standardization relative to gyrA using UA159 as a reference. [file Image3.JPEG]

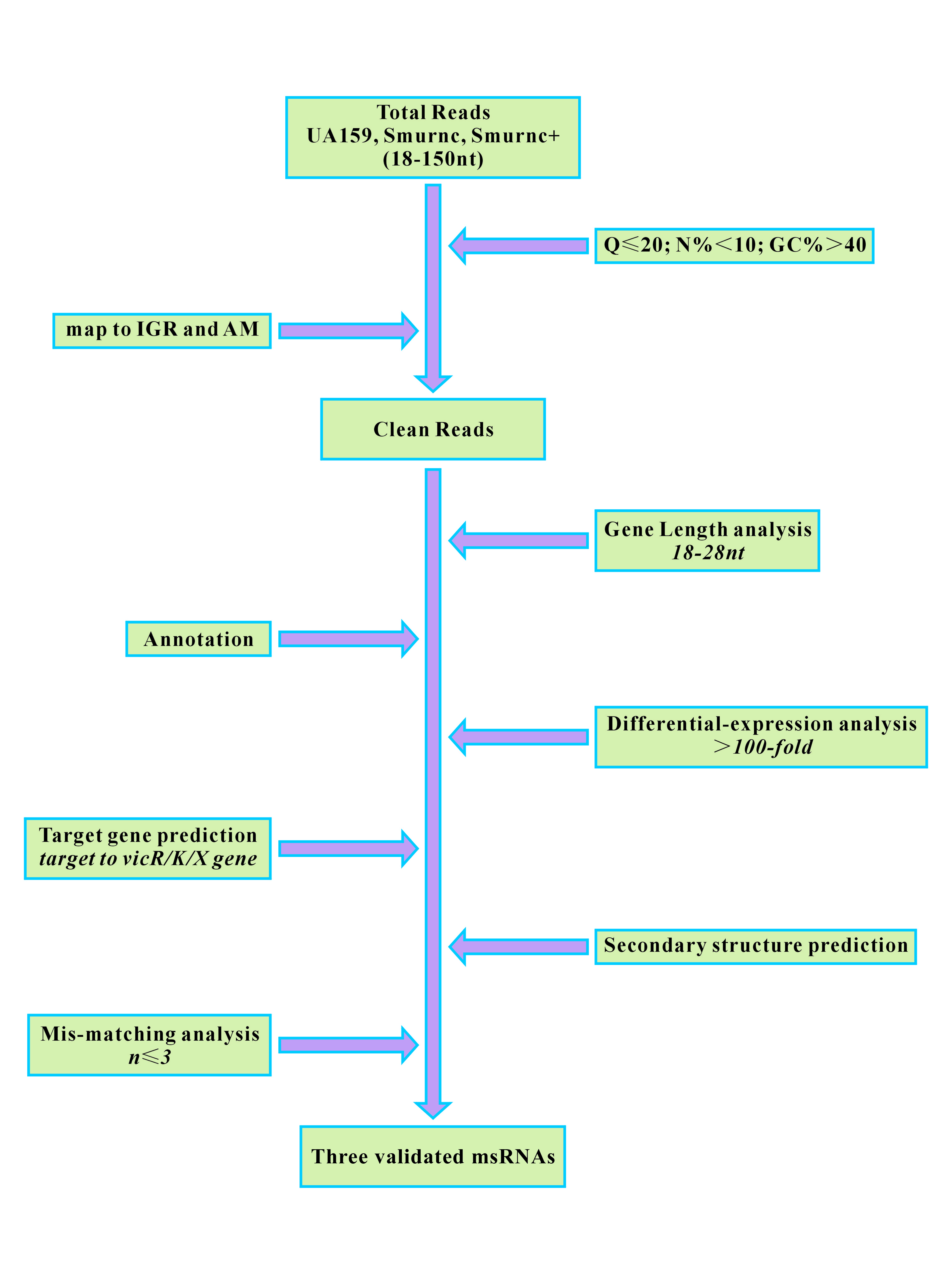

Supplement: Figure S4 — Workflow to analyze and select the validated msRNAs. The directions of the arrows denote the flow of data processing and sequences. The shapes of the boxes have no particular significance, while the descriptions within the boxes represent the steps corresponding to data filtering. [file Image4.JPEG]
