# Supplementary material for: The rnc Gene Promotes Exopolysaccharide Synthesis and Represses the vicRKX Gene Expressions via MicroRNA-Size Small RNAs in Streptococcus mutans
Source: Front Microbiol. 2016 May 10;7:687. doi: 10.3389/fmicb.2016.00687 (PMC4861726; doi:10.3389/fmicb.2016.00687)

The *rnc* Gene Promotes Exopolysaccharide Synthesis and Represses the *vicRKX* Gene Expressions via MicroRNA-Size Small RNAs in *Streptococcus mutans*

Meng-Ying Mao[1,+], Ying-Ming Yang[1,+], Ke-Zeng Li[1,2,+], Lei Lei[1], Meng Li[1], Yan Yang[1], Xiang Tao[3], Jia-Xin Yin[1], Ru Zhang[1,4], Xin-Rong Ma[3, *] & Tao Hu[1,*]

CONSTRUCTION OF SMURNC AND SMURNC^{+} MUTANT STRAINS

S. mutans UA159 was used as the model organism. The resulting *rnc* insertion–deletion mutant was named as Smurnc. The *rnc* gene overexpressed strain was designated as Smurnc^{+}. PCR fragment analyses of Smurnc and Smurnc+ were listed as follows, respectively.

This was for Smurnc. In specific, P1–P2 PCR fragment, WT template (697 bp); erythromycin (erm) cassette PCR confirmation fragment, mutant template (876 bp); P3–P4 PCR fragment, WT template (621 bp); P1–P4 PCR confirmation fragment, mutant template (2194 bp).

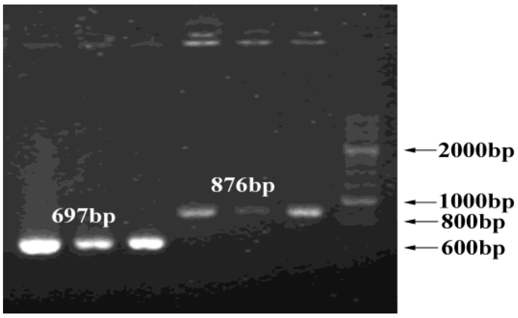

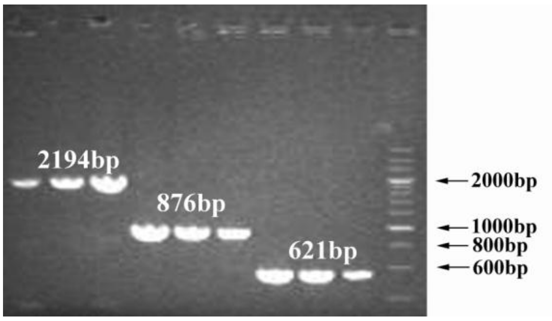

This was for Smurnc^{+}. In specific, *rnc*-F–*rnc*-R PCR confirmation fragment, plasmid extracted from Smurnc^{+} (150 bp).

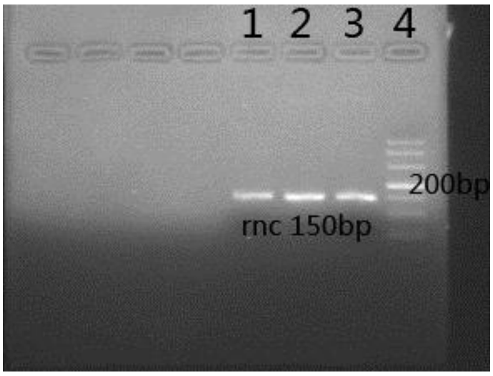

Nucleotide sequence analyses of Smurnc and Smurnc+ were listed as follows, respectively. This was for Smurnc.

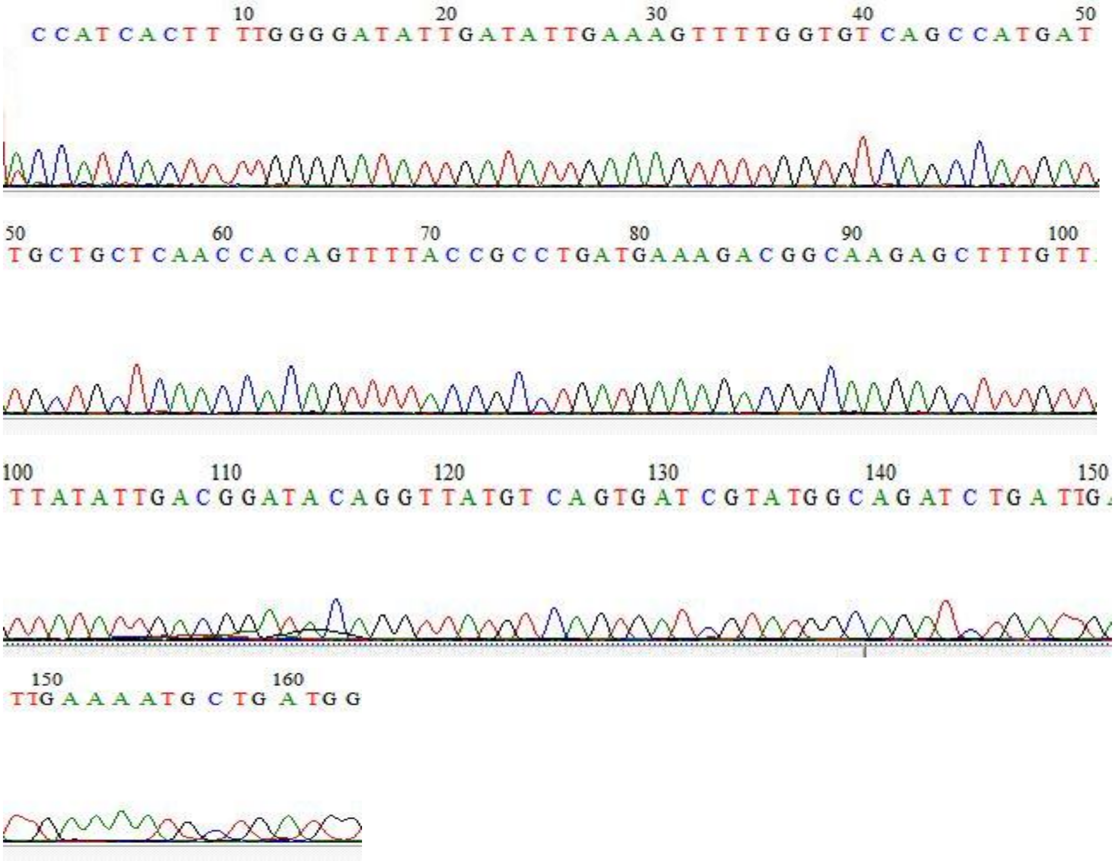

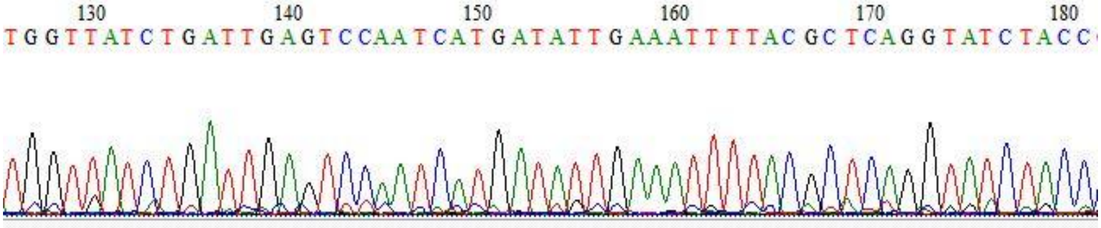

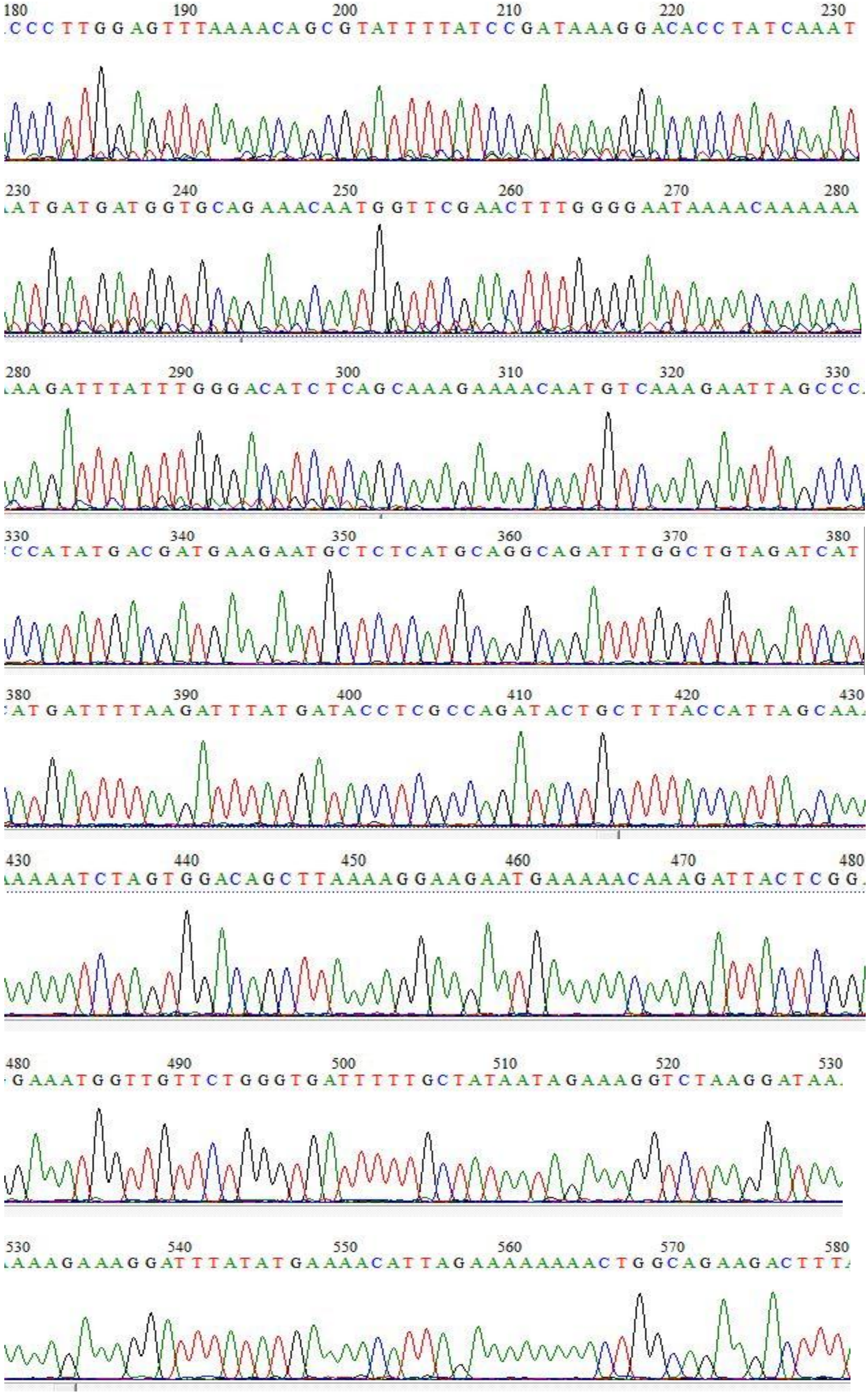
180 190 200 210 220 230
CCCTTGGAGTTTAAAACAGCGTATTTTATCCGATAAAGGACACCTATCAAAT
230 240 250 260 270 280
ATGATGATGGTGCAGAAACAATGGTTCGAACTTTGGGGAATAAAACAAAAAA
280 290 300 310 320 330
AAGATTTATTTGGGACATCTCAGCAAAGAAAACAATGTCAAAGAATTAGCCC
330 340 350 360 370 380
CCATATGACGATGAAGAATGCTCTCATGCAGGCAGATTTGGCTGTAGATCAT
380 390 400 410 420 430
ATGATTTTAAGATTTATGATACCTCGCCAGATACTGCTTTACCATTAGCAA
430 440 450 460 470 480
AAAATCTAGTGGACAGCTTAAAAGGAAGAATGAAAAACAAAGATTACTCGG
480 490 500 510 520 530
GAAATGGTTGTTCTGGGTGATTTTTGCTATAATAGAAAGGTCTAAGGATAA
530 540 550 560 570 580
AAAGAAAGGATTTATATGAAAACATTAGAAAAAAAACTGGCAGAAGACTTT

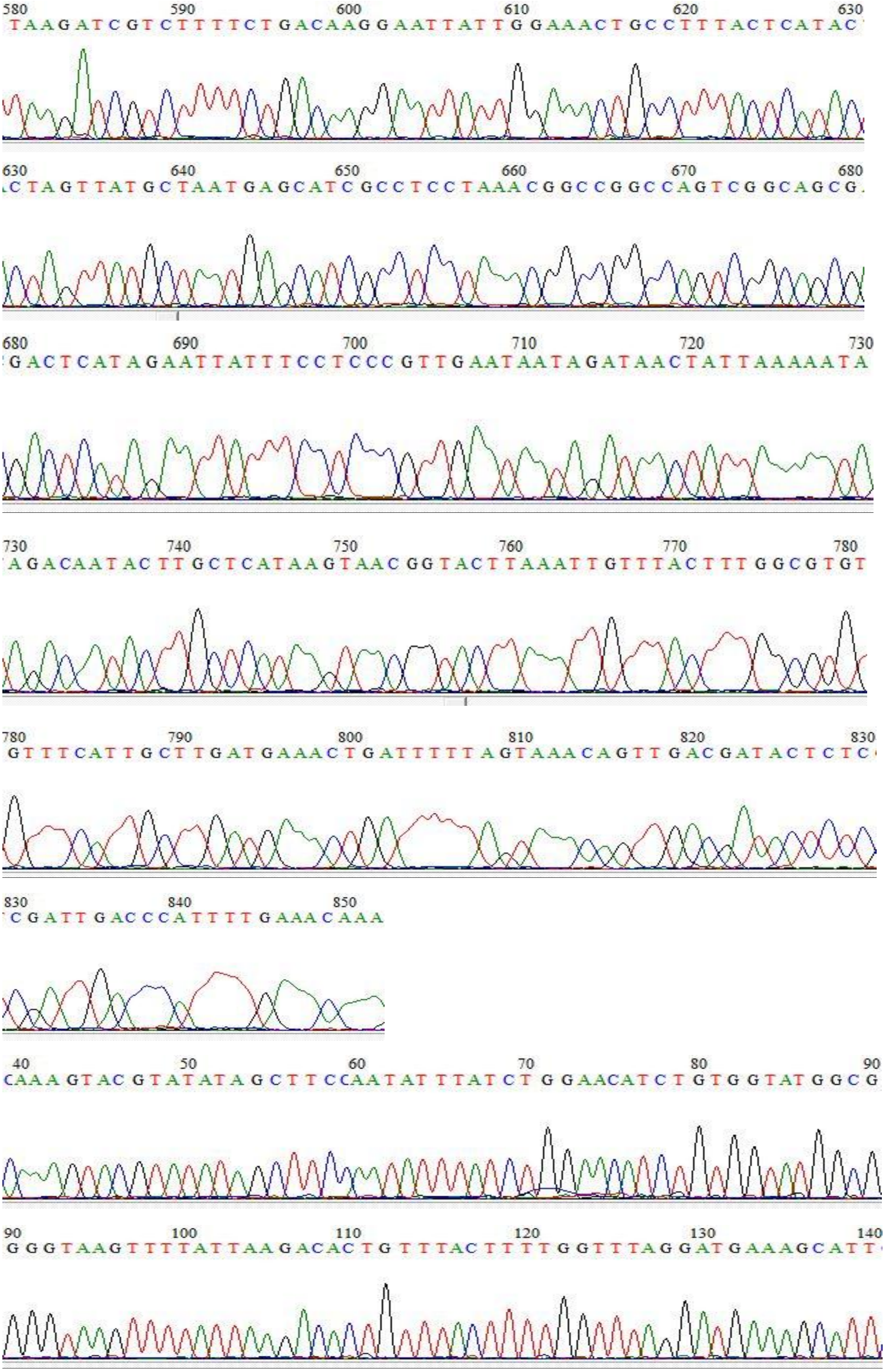
580 590 600 610 620 630
TAAGATCGTCTTTTCTGACAAGGAATTATTGGAAACTGCCTTTACTCATAC
630 640 650 660 670 680
CTAGTTATGCTAATGAGCATCGCCTCCTAAACGGCCGGCCAGTCGGCAGCG
680 690 700 710 720 730
GACTCATAGAATTATTTCCTCCCGTTGAATAATAGATAACTATTAAAAATA
730 740 750 760 770 780
AGACAATACTTGCTCATAAGTAACGGTACTTAAATTGTTTACTTTGGCGTGT
780 790 800 810 820 830
GTTTCATTGCTTGATGAAACTGATTTTTAGTAAACAGTTGACGATACTCTC
830 840 850
CGATTGACCCATTTTGAAACAAA
40 50 60 70 80 90
CAAAGTACGTATATAGCTTCCAATATTTATCTGGAACATCTGTGGTATGGCG
90 100 110 120 130 140
GGGTAAGTTTTATTAAGACACTGTTTACTTTTGGTTTAGGATGAAAGCATT

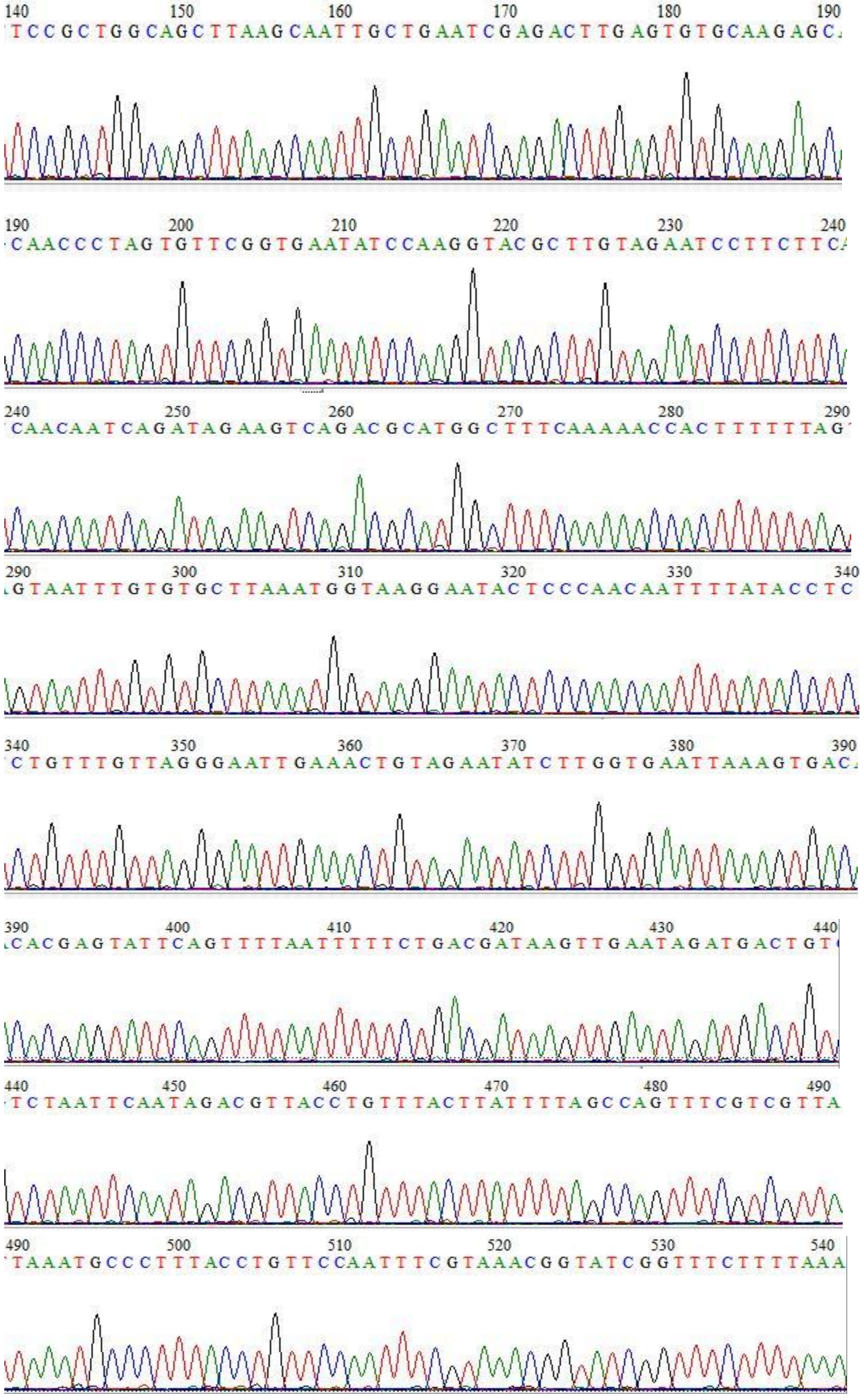
140 150 160 170 180 190
TCCGCTGGCAGCTTAAGCAATTGCTGAATCGAGACTTGAGTGTGCAAGAGC
190 200 210 220 230 240
CAACCCTAGTGTTCGGTGAATATCCAAGGTACGCTTGTAGAATCCTTCTTC
240 250 260 270 280 290
CAACAATCAGATAGAAGTCAGACGCATGGCTTTCAAAAACCACTTTTTTAG
290 300 310 320 330 340
GTAATTTGTGTGCTTAAATGGTAAGGAATACTCCCAACAATTTTATACCTC
340 350 360 370 380 390
CTGTTTGTTAGGGAATTGAAACTGTAGAATATCTTGGTGAATTAAAGTGAC
390 400 410 420 430 440
CACGAGTATTCAGTTTTAATTTTTCTGACGATAAGTTGAATAGATGACTGT
440 450 460 470 480 490
TCTAATTCAATAGACGTTACCTGTTTACTTATTTTAGCCAGTTTCGTCGTTA
490 500 510 520 530 540
TAAATGCCCTTTACCTGTTCCAATTTCGTAAACGGTATCGGTTTCTTTTAAA

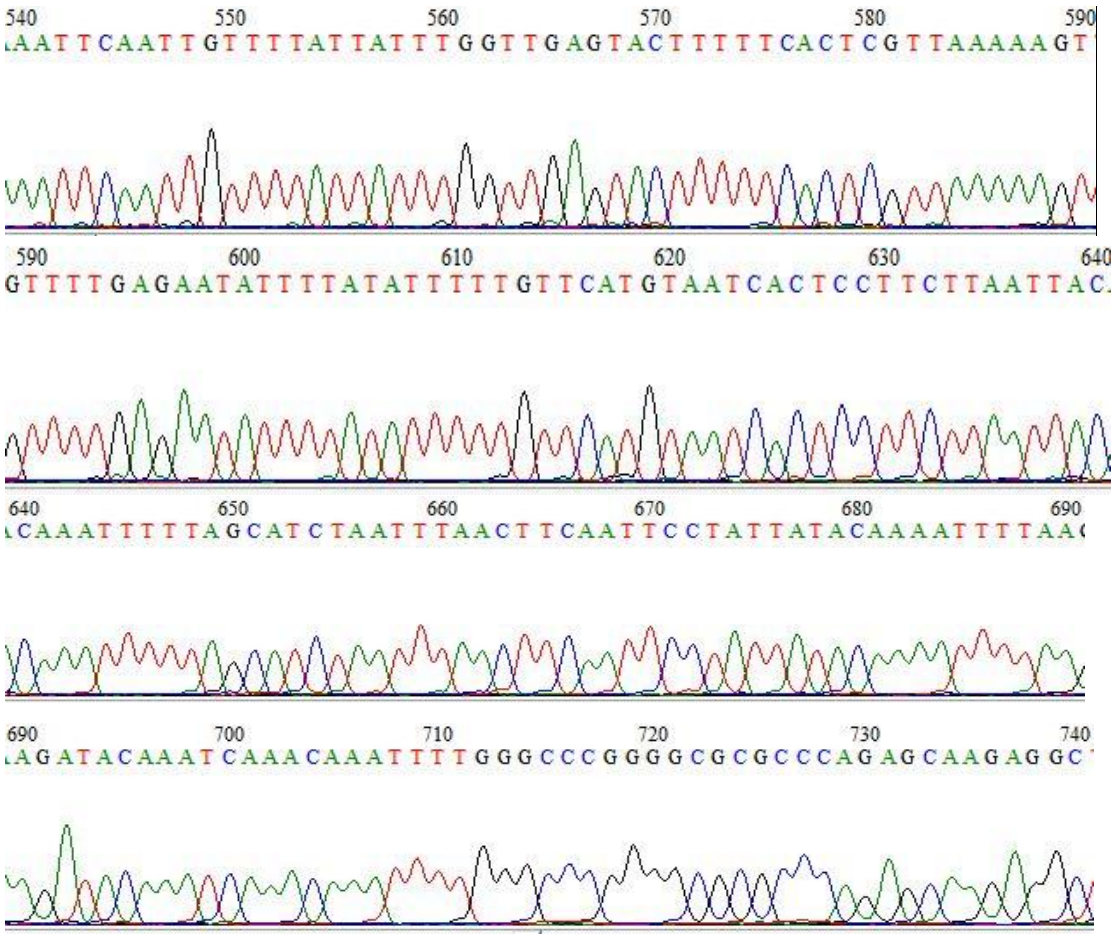
540 550 560 570 580 590
AATTCAATTGTTTTATTATTTGGTTGAGTACTTTTTCACTCGTTAAAAAGT
590 600 610 620 630 640
GTTTTGAGAATATTTTATATTTTTGTTCATGTAATCACTCCTTCTTAATTAC
640 650 660 670 680 690
CAAATTTTTAGCATCTAATTTAACTTCAATTCCTATTATACAAAATTTTAA
690 700 710 720 730 740
AGATACAAATCAAACAAATTTTGGGCCCGGGGCGCGCCCAGAGCAAGAGGC

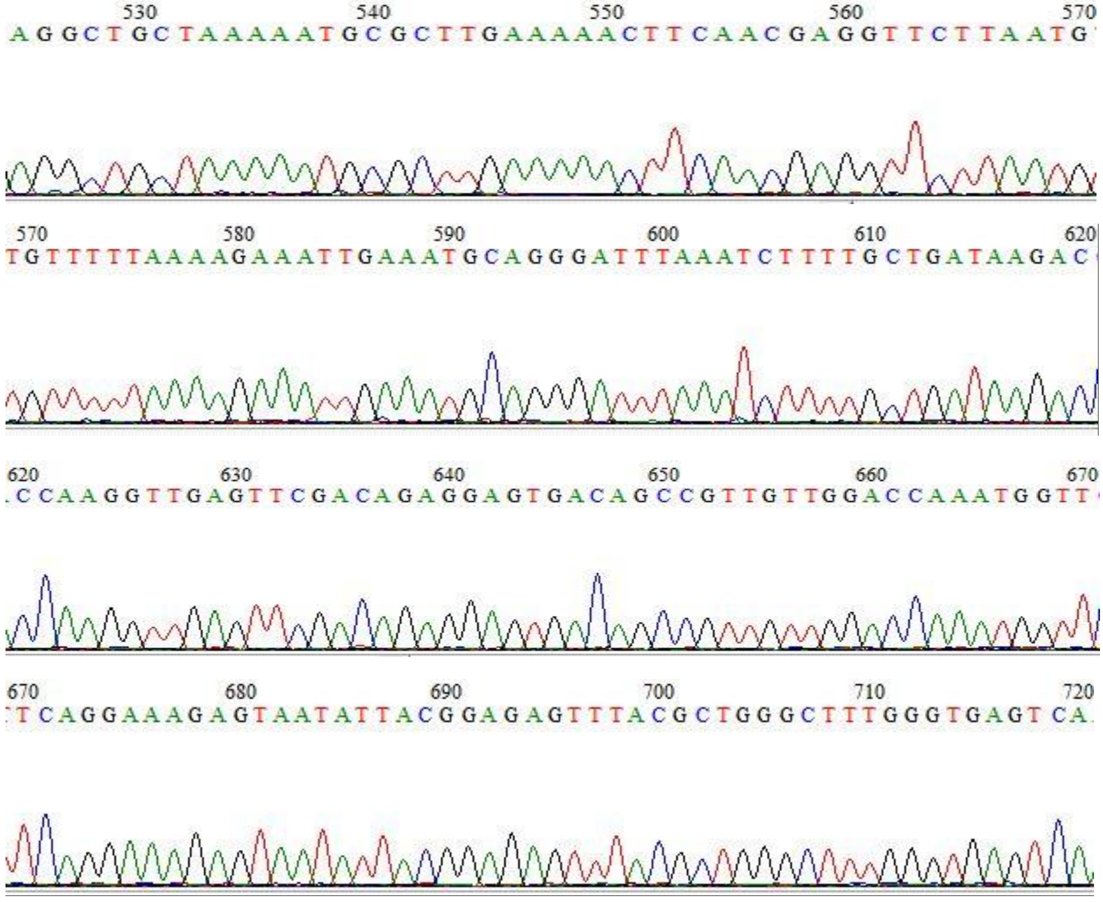
530 540 550 560 570
AGGCTGCTAAAAATGCGCTTGAAAAACTTCAACGAGGTTCTTAATG
570 580 590 600 610 620
TGTTTTTAAAAGAAATTGAAATGCAGGGATTTAAATCTTTTGCTGATAAGAC
620 630 640 650 660 670
CCAAGGTTGAGTTCGACAGAGGAGTGACAGCCGTTGTTGGACCAAATGGTT
670 680 690 700 710 720
TCAGGAAAGAGTAATATTACGGAGAGTTTACGCTGGGCTTTGGGTGAGTCA

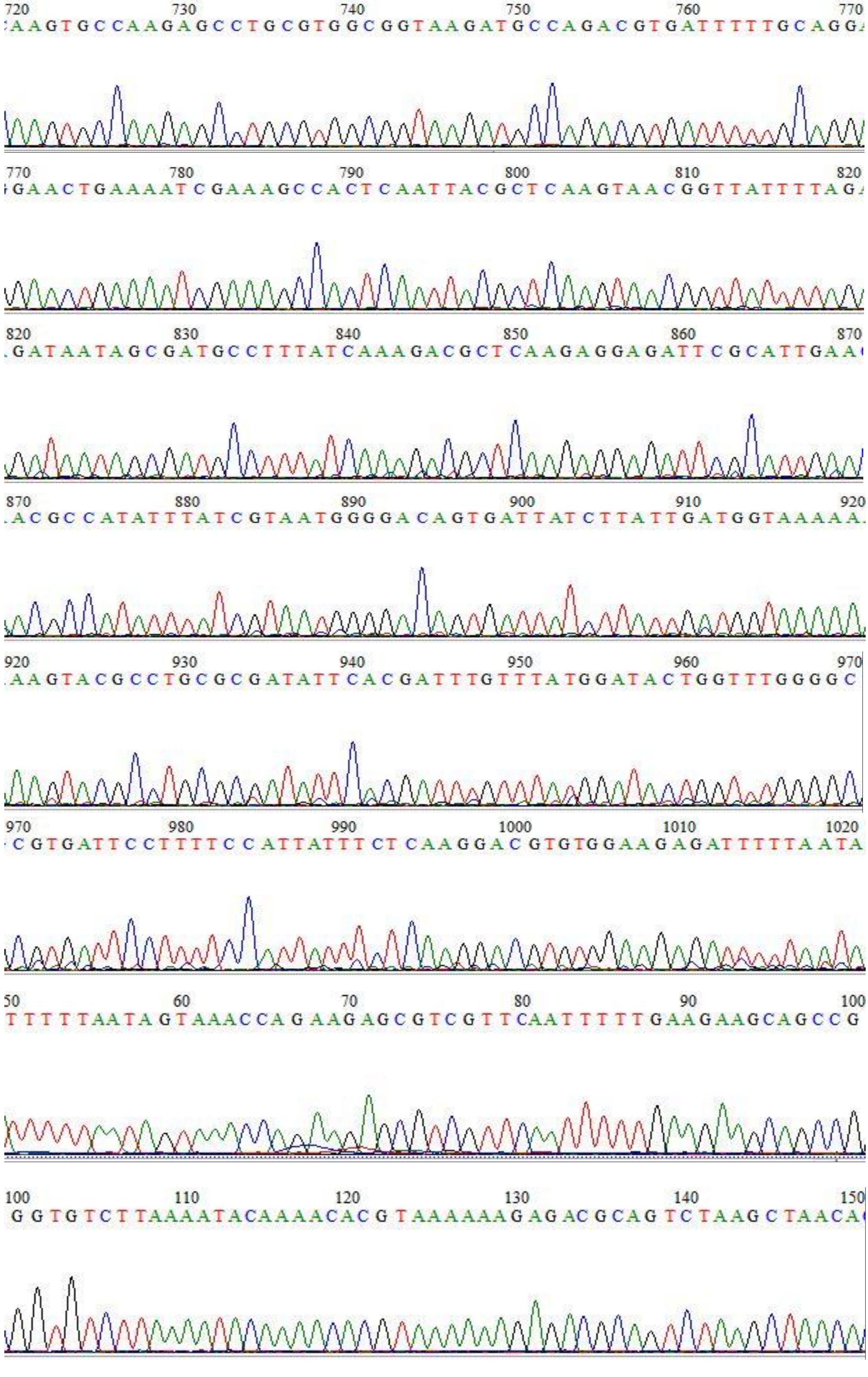
720 730 740 750 760 770
AAGTGCCAAGAGCCTGCGTGGCGGTAAGATGCCAGACGTGATTTTTGCAGGA
770 780 790 800 810 820
GAACTGAAAATCGAAAGCCACTCAATTACGCTCAAGTAACGGTTATTTTAGA
820 830 840 850 860 870
GATAATAGCGATGCCTTTATCAAAGACGCTCAAGAGGAGATTCGCATTGAA
870 880 890 900 910 920
ACGCCATATTTATCGTAATGGGGACAGTGATTATCTTATTGATGGTAAAAA
920 930 940 950 960 970
AAGTACGCCTGCGCGATATTCACGATTTGTTTATGGATACTGGTTTGGGGC
970 980 990 1000 1010 1020
CGTGATTCCTTTTCCATTATTTCTCAAGGACGTGTGGAAGAGATTTTTAATA
50 60 70 80 90 100
TTTTTAATAGTAAACCAGAAGAGCGTCGTTCAATTTTTGAAGAAGCAGCCG
100 110 120 130 140 150
GGTGTCTTAAAATACAAAACACGTAAAAAAGAGACGCAGTCTAAGCTAACA

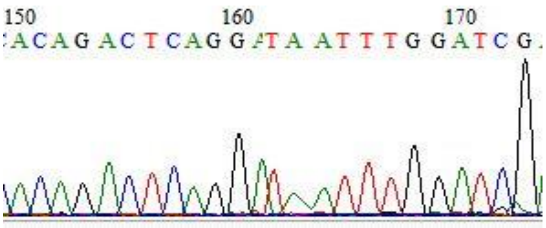

This was for constructed plasmid rncpDL278 extracted from Smurnc+.

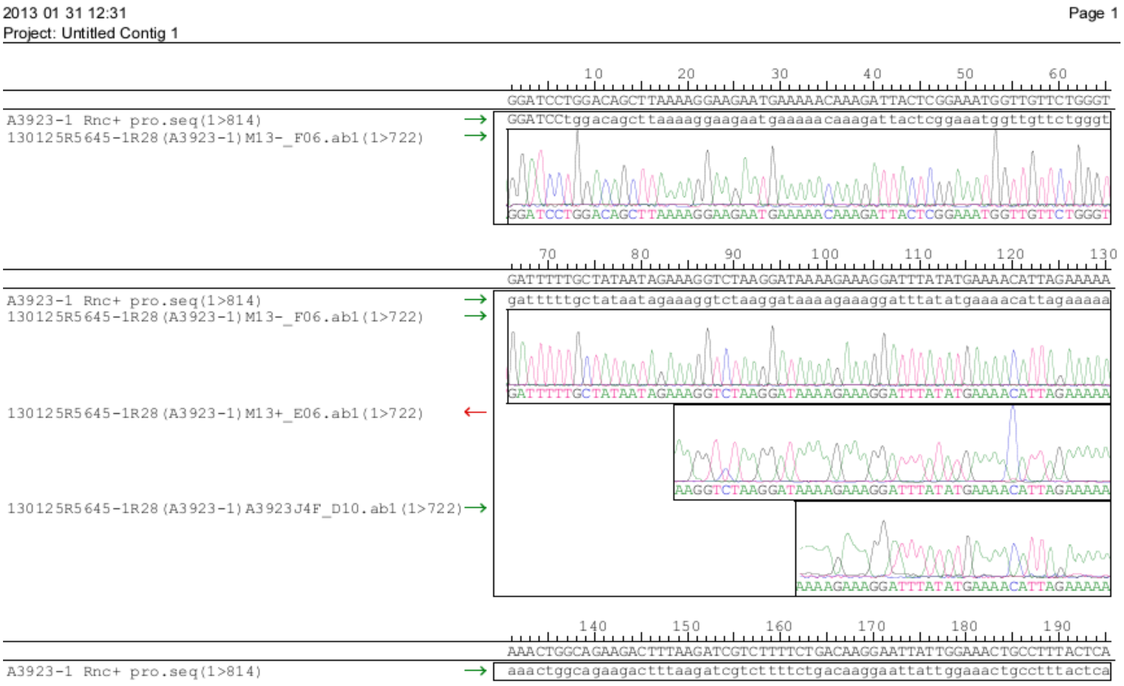

140 150 160 170 180 190

AAACTGGCAGAAGACTTTAAGATCGTCTTTTCTGACAAGGAATTATTGGAAACTGCCTTTACTCA

130125R5645-1R28(A3923-1)M13-_F06.ab1(1>722) →

130125R5645-1R28(A3923-1)M13+_E06.ab1(1>722) ←

130125R5645-1R28(A3923-1)A3923J4F_D10.ab1(1>722) →

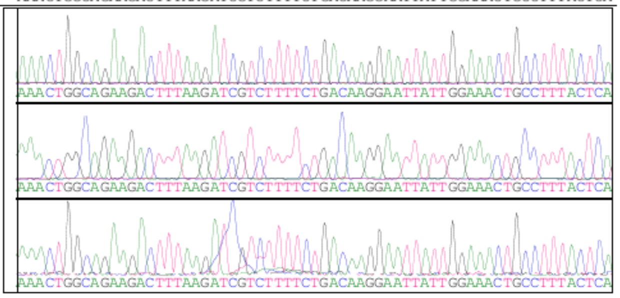

200 210 220 230 240 250 260

TACTAGTTATGCTAATGAGCATCGCCTCCTAAACATTTCACATAACGAGCGCTTGGAATTTTTAG

A3923-1 Rnc+ pro.seq(1>814) → tactagttatgctaatgagcatcgcctcctaaacatttcacataacgagcgcttggaatttttag

130125R5645-1R28(A3923-1)M13-_F06.ab1(1>722) →

130125R5645-1R28(A3923-1)M13+_E06.ab1(1>722) ←

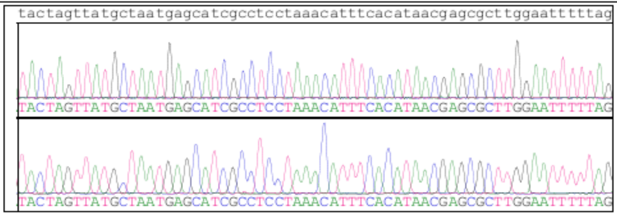

200 210 220 230 240 250 260

TACTAGTTATGCTAATGAGCATCGCCTCCTAAACATTTCACATAACGAGCGCTTGGAATTTTTAG

130125R5645-1R28(A3923-1)A3923J4F_D10.ab1(1>722) →

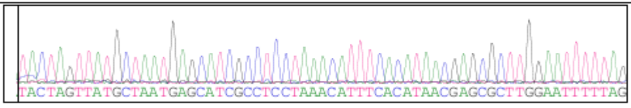

270 280 290 300 310 320

GAGACGCTGTTCTGCAGTTAACGATTTCACATTATCTTTTTGACAAATACCCTCAAAAAGCTGAA

A3923-1 Rnc+ pro.seq(1>814) → gagacgctgttctgcagttaacgatttcacattatctttttgacaaataccctcaaaaagctgaa

130125R5645-1R28(A3923-1)M13-_F06.ab1(1>722) →

130125R5645-1R28(A3923-1)M13+_E06.ab1(1>722) ←

130125R5645-1R28(A3923-1)A3923J4F_D10.ab1(1>722) →

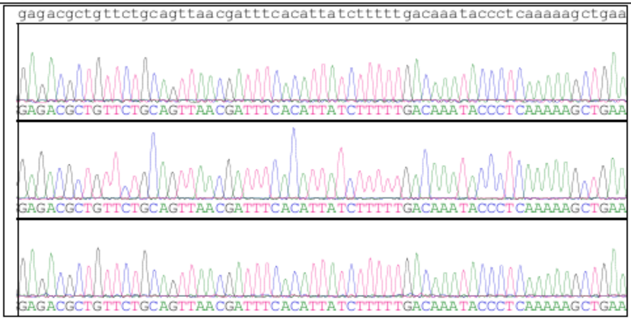

330 340 350 360 370 380 390

GGTGATTTATCAAAAATGCGTTCGATGATTGTTCGTGAAGAAAGTTTGGCGGGTTTTTCTAGAAA

A3923-1 Rnc+ pro.seq(1>814) → ggtgatttatcaaaaatgcgttcgatgattgttcgtgaagaaagtttggcgggtttttctagaaa

130125R5645-1R28(A3923-1)M13-_F06.ab1(1>722) →

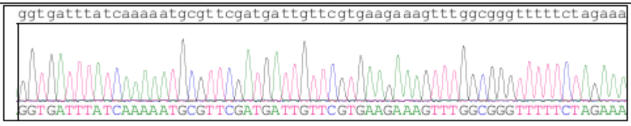

```
        330       340       350       360       370       380       390
GGTGATTTATCAAAAATGCGTTCGATGATTGTTCGTGAAGAAAGTTTGGCGGGTTTTTCTAGAAA
```

130125R5645-1R28(A3923-1)M13+_E06.ab1(1>722) ←

130125R5645-1R28(A3923-1)A3923J4F_D10.ab1(1>722) →

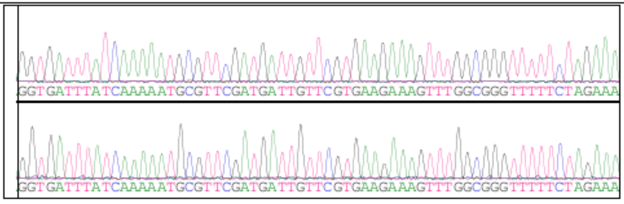


```
          400       410       420       430       440       450
TTGTCACTTTGACCGCTATATTAAATTAGGTAAGGGTGAAGAAAAATCTGGCGGTCGTCAAAGAG
```

A3923-1 Rnc+ pro.seq(1>814) → ttgtcactttgaccgctatattaaattaggtaagggtgaagaaaaatctggcggtcgtcaaagag

130125R5645-1R28(A3923-1)M13-_F06.ab1(1>722) →

130125R5645-1R28(A3923-1)M13+_E06.ab1(1>722) ←

130125R5645-1R28(A3923-1)A3923J4F_D10.ab1(1>722) →

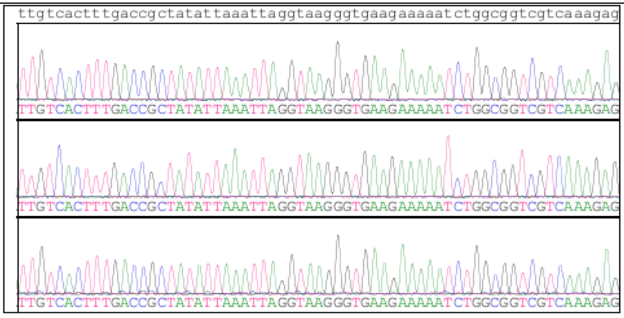


```
        460       470       480       490       500       510       520
ATACTATTTTGGGCGATTTATTTGAGGCTTTTTTAGGTGCTCTTTTATTGGATGCAGGTCTTAAA
```

A3923-1 Rnc+ pro.seq(1>814) → atactattttgggcgatttatttgaggcttttttaggtgctcttttattggatgcaggtcttaaa


```
        460       470       480       490       500       510       520
ATACTATTTTGGGCGATTTATTTGAGGCTTTTTTAGGTGCTCTTTTATTGGATGCAGGTCTTAAA
```

130125R5645-1R28(A3923-1)M13-_F06.ab1(1>722) →

130125R5645-1R28(A3923-1)M13+_E06.ab1(1>722) ←

130125R5645-1R28(A3923-1)A3923J4F_D10.ab1(1>722) →

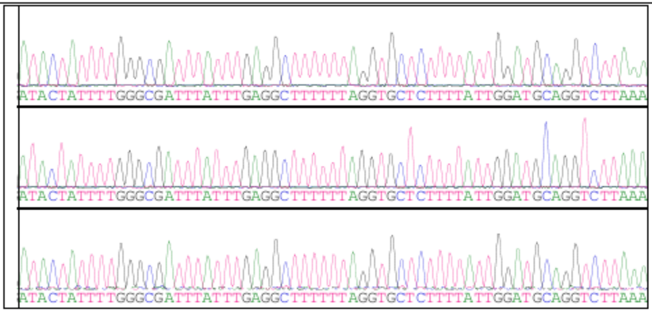


```
          530       540       550       560       570       580
GCTGTTGAGGCCTTTCTCAATCAAGTTGTGATTCCTAAGGTTGAAAATAATAATTATGAACGTGT
```

A3923-1 Rnc+ pro.seq(1>814) → gctgttgaggcctttctcaatcaagttgtgattcctaaggttgaaaataataattatgaacgtgt

130125R5645-1R28(A3923-1)M13-_F06.ab1(1>722) →

130125R5645-1R28(A3923-1)M13+_E06.ab1(1>722) ←

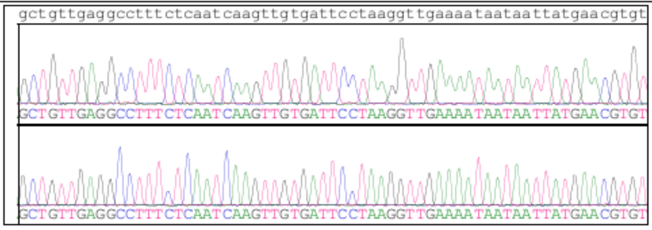

530 540 550 560 570 580

GCTGTTGAGGCCTTTCTCAATCAAGTTGTGATTCCTAAGGTTGAAAATAATAATTATGAACGTGT

130125R5645-1R28(A3923-1)A3923J4F_D10.ab1(1>722) →

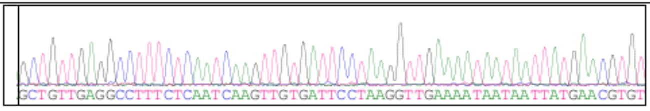

590 600 610 620 630 640 650

TACCGATTATAAAACGGCACTTCAAGAATTATTGCAAGTTGACGGGGATGTTTTGATCGATTACG

A3923-1 Rnc+ pro.seq(1>814) → taccgattataaaacggcacttcaagaattattgcaagttgacggggatgttttgatcgattacg
130125R5645-1R28(A3923-1)M13-_F06.ab1(1>722) →
130125R5645-1R28(A3923-1)M13+_E06.ab1(1>722) ←
130125R5645-1R28(A3923-1)A3923J4F_D10.ab1(1>722) →

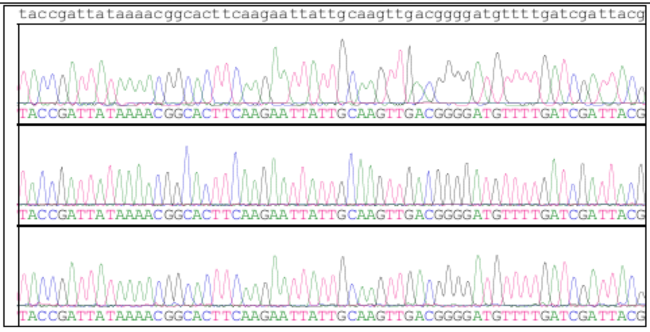

660 670 680 690 700 710

AGGTCTTAAAAGAATCAGGACCCGCCCATGCAAAATGCTTTGAAGTTGCTGTTTCTATGAATCAT

A3923-1 Rnc+ pro.seq(1>814) → aggtcttaaaagaatcaggacccgcccatgcaaaatgctttgaagttgctgtttctatgaatcat
130125R5645-1R28(A3923-1)M13-_F06.ab1(1>722) →

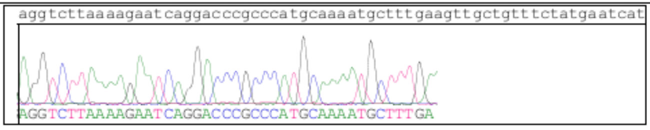

660 670 680 690 700 710

AGGTCTTAAAAGAATCAGGACCCGCCCATGCAAAATGCTTTGAAGTTGCTGTTTCTATGAATCAT

130125R5645-1R28(A3923-1)M13+_E06.ab1(1>722) ←
130125R5645-1R28(A3923-1)A3923J4F_D10.ab1(1>722) →

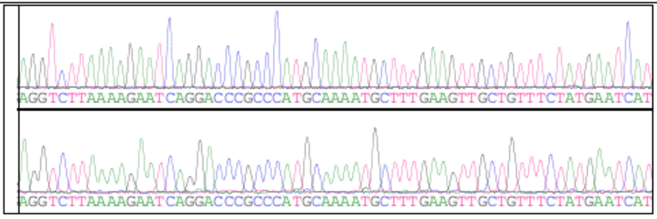

720 730 740 750 760 770 780

GAAAAACTTAGTAGCGGGACAGGCAAATCAAAAAAATTAGCAGAGCAAGAGGCTGCTAAAAATGC

A3923-1 Rnc+ pro.seq(1>814) → gaaaaacttagtagcgggacaggcaaatcaaaaaaattagcagagcaagaggctgctaaaaatgc
130125R5645-1R28(A3923-1)M13+_E06.ab1(1>722) ←
130125R5645-1R28(A3923-1)A3923J4F_D10.ab1(1>722) →

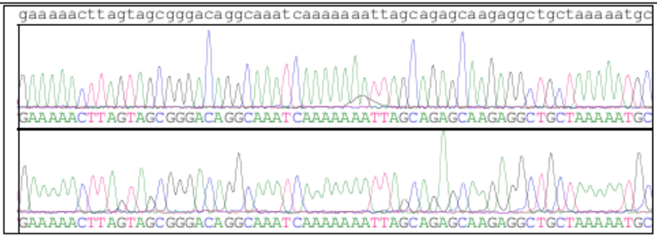

790 800 810 820 830 840

GCTTGAAAAACTTCAACGAGGTTCTTAAGAATTCACTGGCCGTCGTTTTACAACGTCGTGACTGG

A3923-1 Rnc+ pro.seq(1>814) → gcttgaaaaacttcaacgaggttcttaaGAATTC
130125R5645-1R28(A3923-1)M13+_E06.ab1(1>722) ←

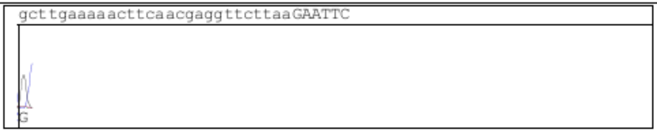

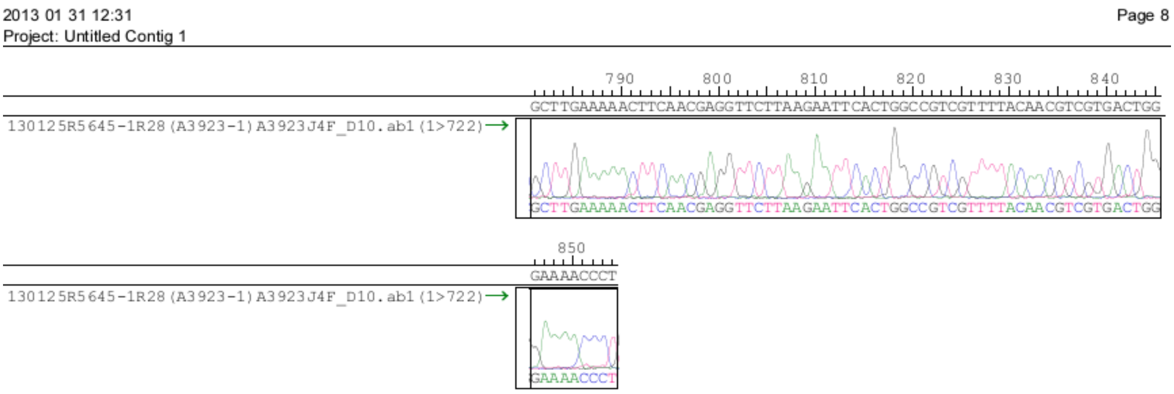

qRT-PCR analyses of *rnc* gene expression in parent UA159 and rnc mutant strains were listed as follow. The fold changes are shown after standardization relative to *gyrA* using UA159 as a reference. The data represent the means and standard errors of the mean values for individuals (n ≥ 3 per group) in three independent experiments. Shapiro－Wilk tests and Bartlett’s tests showed that the data were parametric. Significant differences were determined using ANOVA and two-tailed Student’s t-tests. Asterisks indicate significant differences ($P < 0.05$).

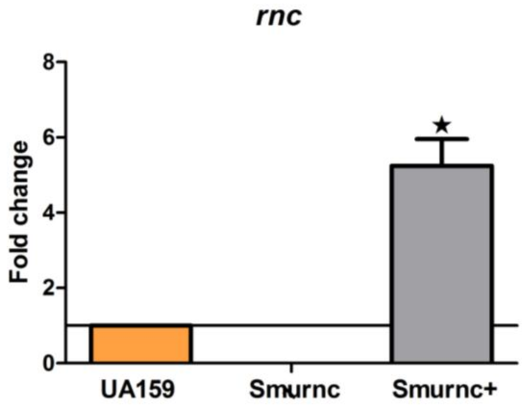

Supplement: Supplementary file 8 [file Presentation1.pdf]
