# Supplementary material for: The rnc Gene Promotes Exopolysaccharide Synthesis and Represses the vicRKX Gene Expressions via MicroRNA-Size Small RNAs in Streptococcus mutans
Source: Front Microbiol. 2016 May 10;7:687. doi: 10.3389/fmicb.2016.00687 (PMC4861726; doi:10.3389/fmicb.2016.00687)
Supplement: Supplementary file 9 [file Presentation2.pdf]

The *rnc* Gene Promotes Exopolysaccharide Synthesis and Represses the *vicRKX* Gene Expressions via MicroRNA-Size Small RNAs in *Streptococcus mutans*

Meng-Ying Mao[1,+], Ying-Ming Yang[1,+], Ke-Zeng Li[1,2,+], Lei Lei[1], Meng Li[1], Yan Yang[1], Xiang Tao[3], Jia-Xin Yin[1], Ru Zhang[1,4], Xin-Rong Ma[3,*] & Tao Hu[1,*]

THE MODIFIED KEYES' SYSTEM

Caries on the sulcal surfaces and its severity was evaluated. The score was determined with some modifications based on the classic and the Larson's Keyes system (Keyes, 1958)(Larson;1981). In particular, we set the severity as three levels, which are E for enamel, Ds for dentin and Dm for 3/4 of the dentin. Precise measurements are not made of depth as general approximations are more than sufficient for comparisons. After checking the infected conditions of mandibular molars, the second molar was most typical. Thus, we took the mandibular second molar as the model to assess the score. The picture showed the scale of units for sulcal lesions.

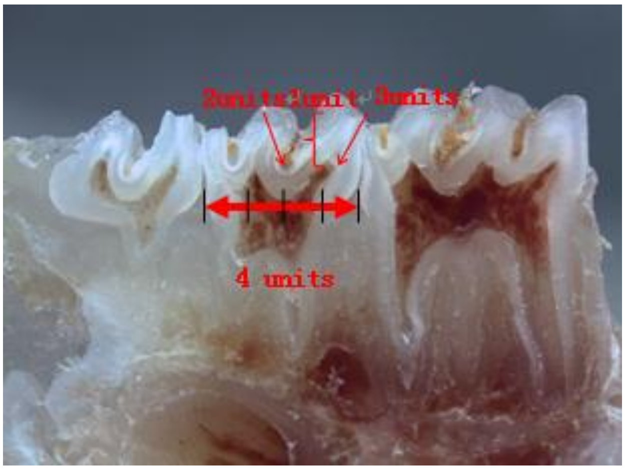

The determination of caries score was blind by codification of the jaws and was done by 1 calibrated examiner. The data were subjected to Kruskal-Wallis test and least significant difference (LSD) multiple comparisons test for all pairs. The level of significance was 5%.

SUPPLRMENTARY MATERIAL REFERENCES

Keyes, P.H. (1958). Dental caries in the molar teeth of rats. II. A method for diagnosing and scoring several types of lesions simultaneously. *J Dent Res* 37**,** 1088-1099.

Larson RM: Merits and modifications of scoring rat dental caries by Keyes' method; in Tanzer JM (ed): Animal Models in Cariology. Microbial Abstr (sp suppl): Washington, IRL Press, 1981, pp 195–203.
